# Effects of caffeine supplementation on post-exercise recovery: a systematic review and three-level meta-analysis of perceptual, functional performance, physiological, and sleep outcomes

**DOI:** 10.3389/fphys.2026.1960237

**Published:** 2026-09-17

**Authors:** Jie Zhang, Yuchao Li, Bo Wei, Xiupeng Li, Zhaonian Xiao, Xiuling Wu, Xing Ye, Shan Zhang

**Affiliations:** 1 School of Physical Education and Tourism, Polus International College, Chengdu, Sichuan, China; 2 Department of Physical Education , University of Electronic Science and Technology of China, Chengdu, Sichuan, China; 3 School of Physical Education, Chengdu Sport University, Chengdu, Sichuan, China; 4 School of Physical Education, Shaanxi Normal University, Xi’an, China; 5 Department of Physical Education, Chengdu Xiangyangqiao Middle School, Chengdu, Sichuan, China; 6 Department of Physical Education, Shanghai Normal University Chengdu Haichuan Middle School, Chengdu, Sichuan, China; 7 School of Sport and Human Movement Science, Beijing Sport University, Beijing, China

**Keywords:** caffeine, functional performance, meta-analysis, muscle damage, muscle soreness, post-exercise recovery, sleep

## Abstract

**Objective:**

To evaluate the effects of caffeine supplementation on perceptual recovery, functional performance during recovery, physiological recovery, and sleep recovery after exercise and explore potential moderators.

**Methods:**

PubMed, Web of Science, Embase, the Cochrane Library, and SPORTDiscus were searched from inception to June 30, 2026, with supplementary searches of Google Scholar, ResearchGate, and reference lists. Randomized controlled trials comparing caffeine with placebo or a caffeine-free condition were included. Hedges’ g was synthesized using three-level random-effects models with REML estimation and CR2 cluster-robust inference. Risk of bias and certainty of evidence were assessed using RoB 2 and GRADE, respectively.

**Results:**

Seventeen studies were included, of which 16 contributed 120 effect sizes to the quantitative synthesis. Caffeine had no clear effect on perceptual recovery (*g* = −0.161, CR2 95% CI −0.528 to 0.205, *p* = 0.319) or physiological recovery (*g* = −0.104, CR2 95% CI −0.346 to 0.138, *p* = 0.293), but produced a small beneficial effect on functional performance during recovery (*g* = −0.208, CR2 95% CI −0.393 to −0.023, *p* = 0.035). Caffeine was associated with poorer sleep recovery (*g* = 0.751, CR2 95% CI 0.244 to 1.258, *p* = 0.018), based on four independent studies. Effects differed across recovery domains (CR2 robust Wald *F*(3, 3.88) = 9.76, *p* = 0.028). Exploratory domain-adjusted moderator analyses were inconclusive for recovery time, caffeine dose, ingestion timing or strategy, and exercise model. Effect directions remained generally stable, but statistical significance for functional performance and sleep varied in sensitivity analyses. Model-based 95% prediction intervals crossed the null for both domains (functional: −0.444 to 0.028; sleep: −0.002 to 1.503). GRADE certainty was very low for perceptual recovery, moderate for functional and physiological recovery, and low for sleep recovery.

**Conclusion:**

Caffeine appears to have domain-specific effects on post-exercise recovery. It may modestly improve functional performance during recovery, does not clearly improve perceptual or physiological recovery, and may impair sleep. Functional and sleep findings should be interpreted cautiously because statistical significance varied in sensitivity analyses and prediction intervals included the null.

**Systematic review registration:**

https://www.crd.york.ac.uk/prospero/display_record.php?ID=CRD420261467699, identifier CRD420261467699.

## 1 Introduction

High-intensity training and competition can deplete energy stores, disrupt muscle structure, and disturb physiological homeostasis, while also inducing muscle soreness, perceived fatigue, and reductions in strength and exercise performance. Effective post-exercise recovery facilitates the restoration of physiological homeostasis, replenishment of energy stores, repair of exercise-induced tissue damage, and maintenance of subsequent training and competitive performance. This is particularly important for athletes facing short recovery intervals or repeated competition demands (Bongiovanni et al., 2020; Naderi et al., 2025). However, post-exercise recovery is not a unitary process. Perceived soreness and fatigue, muscle function, physiological markers of muscle damage, and sleep may follow different recovery trajectories, and the severity of delayed-onset muscle soreness may not accurately reflect the extent of muscle damage (Clarkson and Hubal, 2002; Nosaka et al., 2002). Accordingly, reliance on a single indicator may not adequately capture overall recovery status. Given the multidimensional nature of post-exercise recovery, the present study classified and evaluated relevant outcomes across four domains: perceptual, functional, physiological, and sleep recovery.

Caffeine is one of the most widely used nutritional supplements among physically active individuals and is well established as an acute ergogenic aid, with 3–6 mg/kg body mass being the most commonly investigated effective dose range (Grgic et al., 2018; Guest et al., 2021). Its effects are largely attributed to adenosine-receptor antagonism, which may reduce perceived exertion and pain and help maintain exercise performance. However, such transient improvements in performance do not necessarily indicate accelerated restoration of physiological homeostasis or tissue repair, complicating the interpretation of caffeine as a recovery aid.

The effects of caffeine on perceptual, functional, and physiological recovery after exercise remain controversial. Previous studies have reported inconsistent findings regarding pain and muscle soreness, strength recovery, and physiological markers of muscle damage. Some evidence suggests that caffeine ingestion after exercise-induced muscle damage may reduce pain perception or temporarily improve neuromuscular function; however, these changes are not consistently accompanied by corresponding improvements in physiological markers such as creatine kinase (Atalay et al., 2025; Caldas et al., 2022; Hurley et al., 2013; Maridakis et al., 2007; Muljadi et al., 2021). Existing meta-analyses have also reached different conclusions regarding delayed-onset muscle soreness. Some evidence suggests that caffeine may reduce soreness at specific recovery time points, whereas more recent findings indicate that it does not consistently improve pain at 24, 48, or 72 h after exercise (Atalay et al., 2025; Muljadi et al., 2021). These inconsistencies may reflect differences in exercise-induced muscle damage models, caffeine dose and caffeine ingestion timing, assessment time points, and outcome measures. They also suggest that perceptual improvement, maintenance of functional performance, and physiological tissue repair may represent distinct recovery processes (Caldas et al., 2022; Green et al., 2018; Matsumura et al., 2026; Santos-Mariano et al., 2019).

The effects of caffeine on sleep further complicate its use in post-exercise recovery. Sleep is fundamental to physiological recovery, cognitive function, and training adaptation in athletes, yet caffeine commonly prolongs sleep onset latency, reduces total sleep time and sleep efficiency, and worsens subjective sleep quality (Clark and Landolt, 2017). A systematic review in athletes suggested that these effects may be influenced by dose, caffeine ingestion timing, habitual caffeine consumption, and individual differences in caffeine metabolism (Bodur et al., 2025). A recent meta-analysis further showed that caffeine ingestion before late-afternoon or evening training and competition reduced sleep efficiency and tended to shorten total sleep time (Kocak et al., 2025). Original trials have similarly demonstrated that evening caffeine ingestion may impair sleep architecture, sleep initiation, and subjective recovery the following morning, even when it improves or maintains certain aspects of exercise performance (Ali et al., 2015; Miller et al., 2014; Ramos-Campo et al., 2019). Thus, caffeine use may involve a potential trade-off between short-term functional benefits and impaired sleep recovery.

Previous systematic reviews and meta-analyses have generally focused separately on the acute ergogenic effects of caffeine, delayed-onset muscle soreness, exercise-induced muscle damage, physiological recovery markers, or sleep. Quantitative evidence directly comparing multiple domains of post-exercise recovery within a unified framework remains limited. Moreover, individual studies frequently report multiple recovery outcomes and assessment time points, resulting in dependent effect sizes within the same study that may not be adequately addressed by conventional meta-analytic methods. Therefore, the present systematic review and three-level meta-analysis aimed primarily to quantify the effects of caffeine supplementation on perceptual, functional, physiological, and sleep recovery and to determine whether the effects differed significantly across recovery domains. The overall recovery model and moderator analyses of recovery time, caffeine dose, caffeine ingestion timing, and exercise type were conducted as exploratory analyses.

## 2 Materials and methods

### 2.1 Protocol and registration

This study was conducted in accordance with the Preferred Reporting Items for Systematic Reviews and Meta-Analyses (PRISMA) statement and the *Cochrane Handbook for Systematic Reviews of Interventions*. The review protocol was registered with the International Prospective Register of Systematic Reviews (PROSPERO; registration number: CRD420261467699).

### 2.2 Search strategy

Before the formal literature search, the research team conducted preliminary searches to optimize the combination of search terms and refine the search strategy. Two reviewers independently searched PubMed, Web of Science Core Collection, Embase, the Cochrane Library, and SPORTDiscus via the EBSCOhost platform from database inception to June 30, 2026.

The search strategy combined controlled vocabulary, including Medical Subject Headings (MeSH) and Emtree terms, with free-text terms. It was constructed around four conceptual domains: “caffeine,” “exercise,” “post-exercise recovery,” and “randomized controlled trials.” Terms within each domain were combined using the Boolean operator “OR,” whereas the four domains were combined using “AND.” The main search terms included caffeine, coffee, exercise, training, post-exercise recovery, muscle soreness, fatigue, muscle damage, creatine kinase, sleep, and terms related to randomized controlled trials.

In addition to the electronic database searches, supplementary searches were conducted using Google Scholar and ResearchGate, together with backward citation searching of the reference lists of included studies and relevant systematic reviews and meta-analyses. For transparency and reproducibility, the supplementary searches were documented with respect to the search terms used, search date, and result ordering. Google Scholar and ResearchGate results were screened in order of relevance, and potentially eligible records were assessed according to the same prespecified eligibility criteria used for the database searches. The complete database-specific search strategies and details of the supplementary searches are provided in **Supplementary Table S2**.

### 2.3 Study selection and eligibility criteria

Two reviewers independently screened the records and assessed study eligibility. Full-text articles were evaluated according to the prespecified inclusion and exclusion criteria. Any disagreements regarding study inclusion were resolved through discussion and consensus, with arbitration by a third reviewer when necessary. Reference management and duplicate identification were performed using EndNote 21. The eligibility criteria were developed according to the PICOS framework—Participants, Intervention, Comparator, Outcomes, and Study design—and are described in detail in **Supplementary Table S3**.

### 2.4 Risk of bias assessment

Two reviewers independently assessed the risk of bias in the relevant outcomes of the included studies using the revised Cochrane risk-of-bias tool for randomized trials (RoB 2) (Cochrane, 2021; Sterne et al., 2019). The standard RoB 2 tool was used for randomized parallel-group trials, whereas the RoB 2 tool for crossover trials was applied to randomized crossover studies, with additional assessment of bias arising from period and carryover effects.

Risk-of-bias assessments were conducted at the study-by-recovery-domain level. For studies contributing outcomes to more than one recovery domain, separate RoB 2 judgments were made for perceptual, functional, physiological, and/or sleep recovery, as applicable, rather than assigning a single study-level judgment across all outcomes. Within each study-by-domain assessment, judgments were informed by the outcome measures contributing to that recovery domain. The domains assessed included bias arising from the randomization process, bias due to deviations from intended interventions, bias due to missing outcome data, bias in measurement of the outcome, and bias in selection of the reported result. Each domain and the overall risk of bias were rated as “low risk,” “some concerns,” or “high risk.” Disagreements between the two reviewers were resolved through discussion; when consensus could not be reached, a third reviewer made the final decision.

### 2.5 Data extraction and management

Data from the included studies were extracted and organized using Microsoft Excel^®^ (Microsoft Corporation, Redmond, WA, USA) according to the following categories: (1) study characteristics, including first author, publication year, and study design; (2) participant characteristics, including sample size, age, sex, training background, and sport or exercise discipline; (3) exercise and recovery protocols, including exercise type, intensity, duration, and recovery assessment time points; (4) caffeine intervention characteristics, including the form of supplementation, dose, caffeine ingestion timing, and comparator condition; and (5) perceptual, functional, physiological, and sleep recovery outcomes and their corresponding statistical data. For randomized crossover trials, information on intervention sequence, washout period, and each experimental condition was also extracted.

When a study reported multiple recovery domains, outcome measures, assessment time points, or intervention groups, each was extracted and coded separately. Study identifiers and effect-size identifiers were assigned to preserve the hierarchical structure of effect sizes within studies. Outcome names and units of measurement were standardized during data management. All data were independently extracted and cross-checked by two reviewers. Disagreements were resolved through discussion, with arbitration by a third reviewer when necessary.

### 2.6 Definition and classification of post-exercise recovery outcomes

#### 2.6.1 Definition of post-exercise recovery and outcome domains

In the present study, post-exercise recovery was defined as the process through which exercise-induced perceptual fatigue, reductions in physical function, physiological stress responses, and alterations in sleep progressively return toward pre-exercise levels or a relatively stable state after exercise cessation. Post-exercise recovery is multidimensional, and changes in muscle soreness, physical function, and blood biomarkers do not necessarily occur in parallel; therefore, a single indicator may not adequately reflect overall recovery status (Clarkson and Hubal, 2002; Kellmann et al., 2018; Nosaka et al., 2002). Based on previous research, the outcomes reported in the included studies, and the objectives of the present review, recovery outcomes were classified into the following four domains (Clarkson and Hubal, 2002; Halson, 2014; Kellmann et al., 2018; Nosaka et al., 2002):

1. Perceptual recovery: delayed-onset muscle soreness, pain, perceived fatigue, subjective recovery status, pressure pain threshold, and related outcomes;
2. Functional performance during recovery: maximum voluntary isometric contraction, isokinetic peak torque, jumping, sprinting, endurance performance, and other functional outcomes assessed during the recovery period;
3. Physiological recovery: creatine kinase, lactate dehydrogenase, myoglobin, transaminases, oxidative stress markers, and related physiological indicators;
4. Sleep recovery: total sleep time, sleep efficiency, sleep onset latency, wake after sleep onset, sleep stages, subjective sleep quality, and related outcomes.

When a study reported outcomes from multiple recovery domains or multiple outcome measures, each outcome was extracted and coded separately.

#### 2.6.2 Classification of recovery time and principles for outcome selection

Based on the distribution of assessment time points across the included studies and the prespecified analysis plan, non-sleep recovery outcomes were classified into four time categories: early recovery (≤1 h), >1 to ≤24 h, >24 to ≤48 h, and ≥72 h. Measurements obtained immediately and at 1 h after exercise were combined into the early-recovery category. Outcomes measured specifically at 24 h were classified as >1 to ≤24 h; outcomes measured specifically at 48 h, or reported by the original study as a combined 24–48 h period, were classified as >24 to ≤48 h; and outcomes measured at 72 h or later were classified as ≥72 h. Sleep outcomes were not included in the moderator analysis of recovery time but were categorized according to the assessment period as sleep during the night following exercise or sleep-related assessments conducted the following morning.

Only outcomes intended to reflect the post-exercise recovery process or subsequent sleep were included. Outcomes measured during exercise, or those forming part of the initial exercise task and reflecting only acute exercise performance, training load, or immediate physiological and perceptual responses, were excluded. Examples included repetition number, training volume, heart rate, rating of perceived exertion, and performance during the initial exercise test. Measures such as maximum voluntary contraction, jumping, sprinting, and endurance performance were included only when assessed during the post-exercise recovery period and used to evaluate functional performance during recovery. Because caffeine ingested before exercise may exert residual ergogenic effects on early functional assessments, the timing of early functional outcomes was specifically considered when interpreting the findings (Matsumura et al., 2026). Disagreements regarding outcome classification or assignment of recovery time were resolved through discussion between two reviewers, with arbitration by a third reviewer when necessary.

### 2.7 Effect-size calculation

#### 2.7.1 Effect-size calculation and directional harmonization

Because the included studies assessed post-exercise recovery using outcomes measured on different scales, continuous outcomes were converted to the small-sample bias-corrected standardized mean difference, expressed as Hedges’ g, with the corresponding sampling variance calculated for each effect size (Hedges and Olkin, 1985; Morris and DeShon, 2002). Effect sizes were calculated as the caffeine condition minus the control condition and were directionally harmonized such that g< 0 indicated an effect favoring caffeine, whereas g > 0 indicated an effect favoring the control condition.

For outcomes in which lower values indicated better recovery, such as muscle soreness, fatigue, creatine kinase, sleep onset latency, and wake after sleep onset, the original effect-size direction was retained. For outcomes in which higher values indicated better recovery, such as muscle strength, jump performance, pressure pain threshold, total sleep time, and sleep efficiency, the direction of the effect size was reversed.

#### 2.7.2 Treatment of parallel-group and crossover studies

For randomized parallel-group trials, the standardized mean difference for independent groups was calculated using the means, standard deviations, and sample sizes of the caffeine and control groups. The pooled standard deviation of the two groups was calculated as follows:


$$SD_{pooled}=\sqrt{\frac{\left(n_{CAF}-1\right)SD_{CAF}^{2}+\left(n_{CON}-1\right)SD_{CON}^{2}}{n_{CAF}+n_{CON}-2}}$$

The small-sample bias-corrected Hedges’ g was calculated as follows:


$$g=J\left(\frac{M_{CAF}-M_{CON}}{SD_{pooled}}\right)$$

Where *M_CAF_* and *M_CON_* denote the mean values under the caffeine and control conditions, respectively; *SD_CAF_* and *SD_CON_* denote the corresponding standard deviations; and *J* represents the small-sample bias-correction factor.

For randomized crossover trials, the mean difference between the caffeine and control conditions was standardized using the average variance of the two conditions:


$$SD_{av}=\sqrt{\frac{SD_{CAF}^{2}+SD_{CON}^{2}}{2}}$$

The small-sample bias-corrected effect size was calculated as follows:


$$g_{av}=J\left(\frac{M_{CAF}-M_{CON}}{SD_{av}}\right)$$

The sampling variance for crossover trials accounted for the within-participant correlation between measurements under the caffeine and control conditions (Morris and DeShon, 2002). When the correlation coefficient between conditions was not reported in the original study, a value of *r* = 0.50 was assumed in the primary analysis, and sensitivity analyses were performed to examine the influence of alternative correlation assumptions on the results. Effect sizes from parallel-group and crossover trials were converted to the same metric before inclusion in the meta-analysis, while sampling variances were calculated using methods appropriate to each study design.

#### 2.7.3 Data conversion and extraction of data from figures

When a study reported only the standard error, it was converted to the standard deviation using the following formula:


$$SD=SEM\times \sqrt{n}$$

where *n* denotes the sample size of the corresponding group or experimental condition. When only confidence intervals, test statistics, or other convertible information were reported, these data were transformed into the values required for effect-size calculation according to the methods recommended in the Cochrane Handbook (Higgins et al., 2003, 2024). When means and measures of dispersion were presented only in figures, numerical data were extracted using WebPlotDigitizer and independently verified by two reviewers. Before effect-size calculation, outcome names, measurement units, and effect directions were standardized across studies. The complete effect-level dataset, including Hedges’*g* estimates and corresponding sampling variances for all 120 effect sizes, is provided in the **Supplementary Data**.

### 2.8 Statistical analysis

All statistical analyses were performed in R, primarily using the metafor and clubSandwich packages. Unless otherwise specified, all tests were two-sided, with p< 0.05 considered statistically significant.

#### 2.8.1 Three-level random-effects model and assessment of heterogeneity

Because individual studies could report multiple outcome measures, recovery time points, or intervention conditions, effect sizes derived from the same study were not fully independent. Therefore, a three-level random-effects model was used for the meta-analysis (Cheung, 2014; Viechtbauer, 2010). The model was specified as follows:


$$y_{ij}=\beta_{0}+u_{j}+w_{ij}+\epsilon_{ij}$$

where *y_ij_* denotes the *i*th effect size from the *j*th study, *β*_0_ represents the overall mean effect, *u_j_* denotes the between-study random effect, *w_ij_* represents the random variation among effect sizes within the same study, and *ϵ_ij_* denotes the sampling error with variance *v_ij_*. The model specified sampling error at Level 1, variation among effect sizes within the same study at Level 2, and between-study variation at Level 3. Variance components were estimated using restricted maximum likelihood (REML). The random-effects structure specified effect sizes as nested within studies.

To reduce the influence of within-study effect-size dependence and the limited number of independent studies on statistical inference, CR2 cluster-robust variance estimation with small-sample degrees-of-freedom corrections was applied, using study as the clustering unit (Tipton and Pustejovsky, 2015). Unless otherwise specified, results are reported as pooled Hedges’ g. both 95% confidence intervals and p values were obtained using CR2 robust inference with study as the clustering unit and Satterthwaite small-sample degrees-of-freedom corrections, whereas 95% prediction intervals were estimated from the three-level random-effects models and reported separately as model-based quantities. Heterogeneity was evaluated using Cochran’s *Q* test, the Level 2 and Level 3 variance components, and the total *I*^2^ and its decomposition across the two levels (Cheung, 2014; Higgins et al., 2003).

#### 2.8.2 Primary and exploratory analyses

For the primary analyses, separate three-level random-effects models were fitted for perceptual, functional, physiological, and sleep recovery to estimate the pooled effect of caffeine within each recovery domain. Statistical inference was conducted independently for each domain, and the overall recovery result was not used as a substitute for domain-specific findings. In addition, all recovery outcomes were included in a single model to explore the overall pooled effect of caffeine on post-exercise recovery. Because the direction and practical meaning of effects may differ across recovery domains, the overall recovery model was considered a supplementary analysis and was not used as the basis for the primary conclusions.

#### 2.8.3 Moderator analyses, subgroup analyses, and meta-regression

Potential moderators were examined using mixed-effects three-level meta-regression models estimated with restricted maximum likelihood, with effect sizes nested within studies as the random-effects structure. All moderator analyses used study as the clustering unit and applied CR2 cluster-robust variance estimation. For individual regression coefficients and subgroup-specific effect estimates, 95% confidence intervals and p values were obtained using CR2 robust inference with Satterthwaite small-sample degrees-of-freedom corrections. The overall effects of multi-parameter categorical moderators were evaluated using robust Wald tests using the approximate Hotelling’s T²_Z_ (HTZ) small-sample correction, with the corresponding numerator and denominator degrees of freedom reported.

Recovery domain was first entered as a categorical moderator to test the overall differences among perceptual, functional, physiological, and sleep recovery. Where data permitted, the potential moderating effects of recovery time, caffeine dose, ingestion timing or strategy, and exercise type or recovery model were further explored. Given the differences in effects across recovery domains, recovery domain was included as a covariate in moderator analyses that pooled outcomes across multiple recovery domains, thereby providing domain-adjusted moderator estimates. Categorical moderators were entered as dummy variables, and domain-adjusted effect estimates for each level together with the overall moderator test were reported. Caffeine dose was evaluated using both categorical subgroup analysis and continuous-dose meta-regression. Interactions between recovery domain and individual moderators were explored only when the distribution of independent studies across the corresponding domain-by-moderator combinations was sufficient to support such analyses. All moderator analyses were considered exploratory and were interpreted cautiously in light of the number of independent studies within each level, the width of the CR2 confidence intervals, and the stability of the estimates (Cheung, 2014; Tipton and Pustejovsky, 2015).

#### 2.8.4 Sensitivity analyses

To address uncertainty in the assumed within-participant correlation for randomized crossover trials, in addition to the primary assumption of *r* = 0.50, alternative values of *r* = 0.30 and *r* = 0.70 were used to recalculate the sampling variances of effect sizes from crossover trials, and the corresponding models were refitted to evaluate the influence of the correlation assumption on the results. Leave-one-study-out analyses were then conducted by excluding one study at a time and refitting the models to assess the influence of individual studies on the direction, magnitude, and statistical inference of the pooled effects. In addition, based on the study-by-recovery-domain RoB 2 assessments, studies judged to be at high risk of bias for a specific recovery domain were excluded within that domain, and the corresponding domain-specific models were refitted to evaluate the robustness of the primary findings to risk of bias.

To examine whether studies contributing multiple outcomes or time points exerted disproportionate influence on the pooled estimates, an additional one-study-one-domain sensitivity analysis was conducted. Within each study and recovery domain, all eligible effect sizes were combined into a single equal-weighted composite effect. A conservative working correlation of ρ = 1.00 was assumed among effect sizes within each study-by-domain combination when calculating the sampling variance of the composite effect, thereby avoiding artificial gains in precision from denser outcome or time-point reporting. The resulting study-level composite effects were then pooled using random-effects meta-analysis with REML estimation and CR2 robust inference.

Because functional outcomes assessed shortly after caffeine ingestion may reflect acute or residual ergogenic effects rather than accelerated recovery, additional sensitivity analyses were conducted for the functional domain by excluding assessments performed within 1 h after exercise, excluding outcomes for which caffeine was ingested specifically before the recovery test, and applying both restrictions simultaneously. Detailed results are presented in **Supplementary Table S4**.

#### 2.8.5 Assessment of small-study effects and publication bias

To evaluate potential small-study effects and funnel-plot asymmetry, a domain-adjusted three-level Egger regression was first performed using the overall effect-level dataset, with the standard error of the effect size and recovery domain entered as moderators. Regression coefficients were estimated using CR2 cluster-robust variance estimation with study as the clustering unit, together with Satterthwaite small-sample degrees-of-freedom correction. For descriptive visualization, contour-enhanced funnel plots were constructed separately for each recovery domain using one study-level composite effect per study and domain. When a study contributed multiple eligible effect sizes within the same domain, these were combined into an equal-weight composite, with the sampling variance calculated under a conservative within-study correlation of ρ = 1.00. These study-level plots were used only for visual assessment and did not replace the effect-level three-level Egger regression.

Exploratory three-level Egger regressions were then conducted separately for the perceptual recovery, functional performance during recovery, physiological recovery, and sleep recovery domains. Domain-specific contour-enhanced and power-enhanced funnel plots were additionally examined as exploratory diagnostics. For each power-enhanced funnel plot, the pooled effect from the corresponding domain-specific three-level model was used as the assumed true effect to illustrate theoretical statistical power across different levels of standard error and the distribution of effect sizes with low statistical power (Egger et al., 1997; Kossmeier et al., 2020; Peters et al., 2008). Because fewer than 10 independent studies were included in each recovery domain, the domain-specific findings were interpreted as exploratory and were not considered definitive evidence regarding the presence or absence of small-study effects (Higgins et al., 2003). These analyses were not used to rule out publication bias in the GRADE assessment. Detailed results are provided in **Supplementary Table S5**.

### 2.9 Certainty of evidence assessment

The certainty of evidence for the four primary recovery domains—perceptual, functional, physiological, and sleep recovery—was assessed using the GRADE (Grading of Recommendations Assessment, Development and Evaluation) approach (Guyatt et al., 2008; Schünemann et al., 2013). Because all included studies were randomized controlled trials, the certainty of evidence for each recovery domain was initially rated as high and was subsequently downgraded, where appropriate, across five domains: risk of bias, inconsistency, indirectness, imprecision, and publication bias. Final certainty ratings were classified as high, moderate, low, or very low.

Risk of bias was evaluated on the basis of the study-by-recovery-domain RoB 2 assessments, together with sensitivity analyses excluding studies judged to be at high risk of bias within the corresponding recovery domain, in order to determine whether risk of bias was likely to materially influence the pooled effect estimate.

Inconsistency was evaluated by considering between-study and within-study heterogeneity, the consistency of effect directions across studies, and the 95% prediction interval. Downgrading for inconsistency was considered when substantial heterogeneity was present, when effect directions varied meaningfully across studies, or when the prediction interval encompassed effects with materially different interpretations.

Indirectness was assessed according to the extent to which the populations, caffeine interventions, comparator conditions, and outcome measures in the included studies corresponded to the prespecified review question.

Imprecision was not judged using a single fixed standardized-effect threshold across all recovery domains. Instead, it was assessed by considering the number of independent studies, the number of unique participants contributing to each recovery domain, the width and range of the CR2 robust 95% confidence interval, and the stability of the findings across sensitivity analyses. Downgrading for imprecision was considered when confidence intervals were wide and encompassed effects with meaningfully different interpretations, such as beneficial, near-null, and adverse effects, or when the limited number of independent studies and participants resulted in substantial uncertainty in the pooled estimate. The 95% prediction interval was interpreted as complementary information regarding the range of potential true effects in future comparable studies and was reported separately from the confidence interval.

Publication bias was judged by integrating the number of available studies, funnel-plot patterns, and the findings of three-level Egger regression. Because fewer than 10 independent studies were available within each recovery domain, domain-specific funnel plots and Egger regression results were considered exploratory. A non-significant Egger test was not regarded as sufficient evidence to rule out publication bias, and for domains with very few independent studies, the presence or absence of small-study effects could not be determined reliably from these statistical methods alone.

Two reviewers independently completed the GRADE assessments, and disagreements were resolved through discussion. The exploratory overall model, subgroup analyses, and meta-regression analyses were not assessed using GRADE. Detailed evidence profiles and reasons for downgrading are presented in **Supplementary Table S6**.

## 3 Results

### 3.1 Study selection

The electronic database searches identified 2,918 records, including 1,379 from Web of Science Core Collection, 713 from Embase, 222 from PubMed, 443 from the Cochrane Library, and 161 from SPORTDiscus. After removal of 619 duplicate records, 2,299 records remained for title and abstract screening. Of these, 1,958 records were excluded, leaving 341 full-text reports for eligibility assessment. Following full-text assessment, 325 reports were excluded because of an ineligible intervention (n = 82), comparator (n = 78), outcome (n = 71), or study design (n = 64), or because of insufficient data or reporting (n = 30). Consequently, 16 studies were included from the database searches.

In addition, 18 records were identified through other methods, including Google Scholar, ResearchGate, and citation searching of reference lists. After screening, 10 records were excluded, and eight reports were assessed for eligibility. Seven reports were subsequently excluded because of an ineligible intervention (n = 4), study design (n = 2), or outcome (n = 1), resulting in one additional study being included. Overall, 17 studies were included in the systematic review, of which 16 contributed to the quantitative meta-analysis and one was included in the qualitative synthesis only. The study selection process is presented in **Figure 1**.

**Figure 1:**
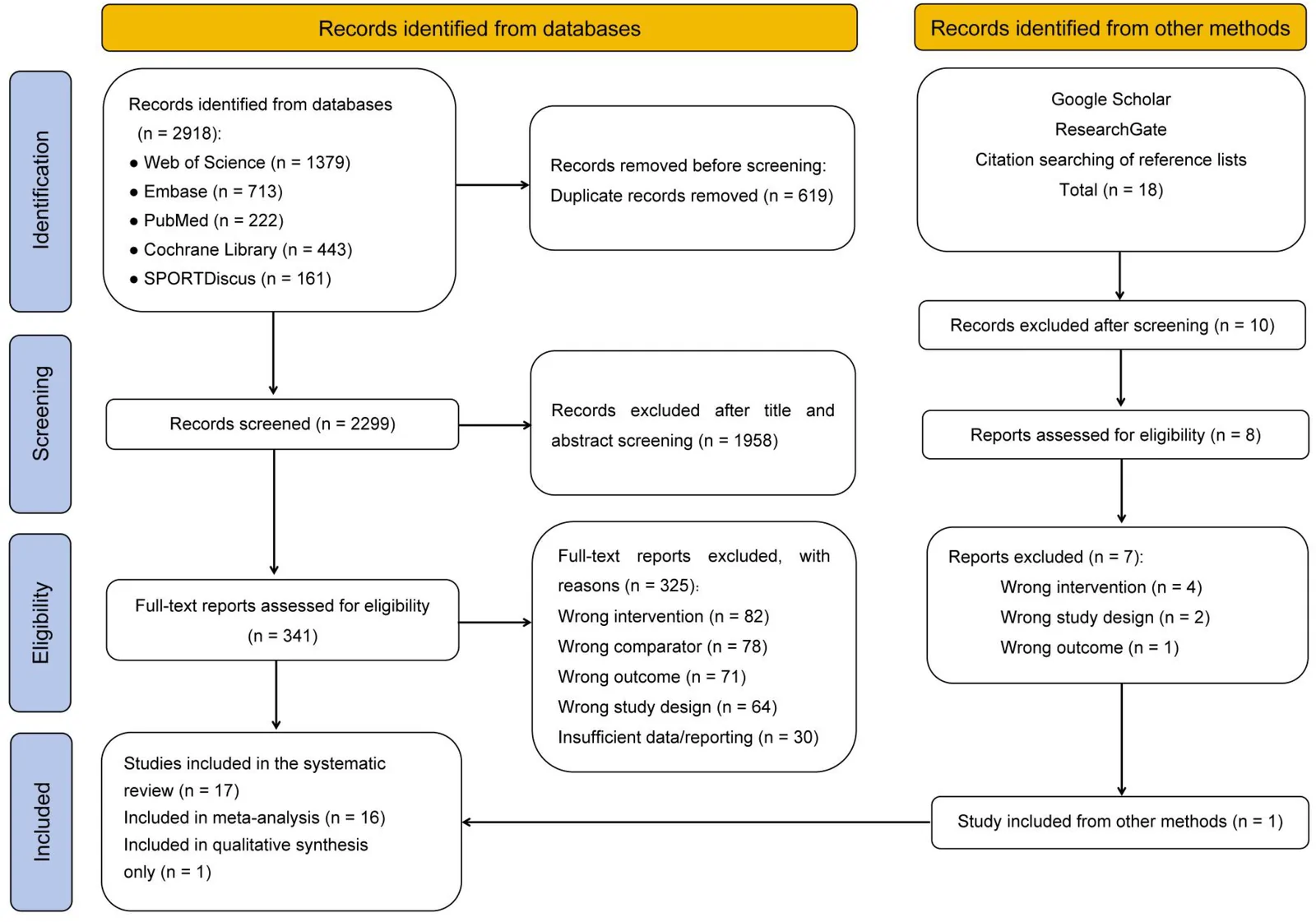
PRISMA flow diagram of the study selection process.

### 3.2 Characteristics of the included studies

Seventeen studies published between 2007 and 2025 were included, comprising 268 unique participants, with individual study sample sizes ranging from 6 to 54 participants. Of these, 214 participants from 16 studies contributed to the quantitative meta-analysis. Fourteen studies used randomized crossover designs, whereas three used parallel-group designs. Participants were predominantly athletes, trained individuals, or physically active healthy adults. Most were young men, although several studies included both men and women.

The exercise and recovery models primarily included muscle damage induced by resistance or eccentric exercise, running- and cycling-based endurance exercise, intermittent sprint exercise, and sport-specific activities such as football and judo. Several studies also assessed sleep following evening exercise and recovery the following morning. Caffeine was most commonly administered in capsule or tablet form, with doses generally ranging from 3 to 6 mg/kg. It was typically ingested 30–60 min before exercise or a recovery assessment, although a small number of studies used post-exercise administration or repeated ingestion during the recovery period. Recovery outcomes were assessed from immediately after exercise to 120 h post-exercise, whereas sleep outcomes were evaluated primarily during the night following exercise and the subsequent morning.

The included outcomes covered all four recovery domains: perceptual, functional, physiological, and sleep recovery. One study used a four-group balanced placebo design, from which a caffeine-versus-placebo effect size that was consistent and comparable with those of the other studies could not be derived using the prespecified methods; therefore, this study was included only in the qualitative synthesis (Bezuglov et al., 2025). The remaining 16 studies were included in the quantitative meta-analysis. Detailed characteristics of the included studies are presented in **Supplementary Table S7** (AbuMoh'd et al., 2021; Al-Nawaiseh et al., 2022; Ali et al., 2015; Bezuglov et al., 2025; Caldwell et al., 2017; Chen et al., 2019; Filip-Stachnik, 2022; Filip-Stachnik et al., 2023; Green et al., 2018; Hurley et al., 2013; MaChado et al., 2009, 2008; Maridakis et al., 2007; Miller et al., 2014; Park et al., 2008; Ramos-Campo et al., 2019; Santos-Mariano et al., 2019).

### 3.3 Risk of bias assessment

Risk of bias was reassessed at the study-by-recovery-domain level. Among the studies contributing to the quantitative synthesis, there were 25 study-by-domain assessments across the four recovery domains. Overall, 19 assessments (76.0%) were rated as having some concerns and 6 (24.0%) as being at high risk of bias; none were rated as low risk overall. Within perceptual recovery, 3 of 7 study-level domain assessments were rated as high risk and 4 as having some concerns. Within the functional-performance domain, 2 of 9 were rated as high risk and 7 as having some concerns. Within physiological recovery, 1 of 5 was rated as high risk and 4 as having some concerns. All 4 studies contributing to sleep recovery were rated as having some concerns, with none rated as high risk. The study included only in the qualitative synthesis (Bezuglov et al., 2025) was rated as having some concerns for both the perceptual and physiological recovery domains.

The main sources of potential bias included insufficient reporting of the randomization process, period or carryover concerns in some crossover trials, missing outcome data, measurement-related concerns particularly for subjective outcomes, and potential selective reporting. Detailed study-by-recovery-domain RoB 2 assessments are presented in **Supplementary Figures S2A**, **S2B** and **Supplementary Tables S8A**, **S8B**.

### 3.4 Effects of caffeine on post-exercise recovery

#### 3.4.1 Effects of caffeine on perceptual recovery

As shown in **Figure 2**, seven studies involving 86 unique participants contributed 26 effect sizes for perceptual recovery, primarily involving post-exercise muscle soreness, pain, fatigue, and subjective recovery status. The three-level random-effects model showed that the pooled effect slightly favored caffeine but was not statistically significant (Hedges’*g* = −0.161, CR2 95% CI: −0.528 to 0.205, CR2 *p* = 0.319).

**Figure 2:**
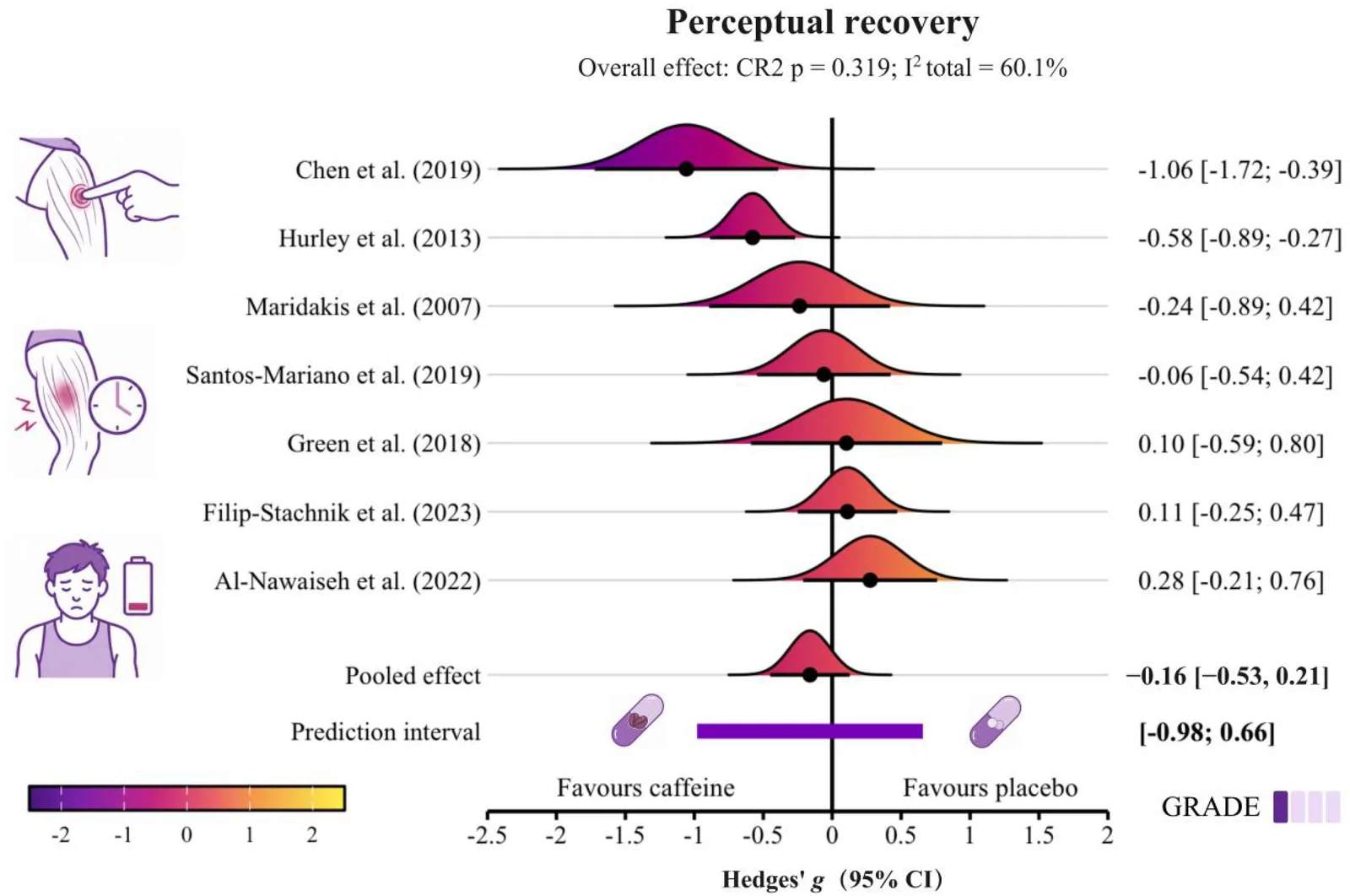
Distribution-enhanced forest plot of the effects of caffeine supplementation on perceptual recovery after exercise. Negative effect sizes favor caffeine, whereas positive values favor placebo. For visualization, multiple effect sizes from the same study within a recovery domain were combined using inverse-variance weighting to obtain a study-level Hedges’*g* and standard error; these summaries were used only for graphical display, whereas pooled estimates were based on all effect-level data in the three-level random-effects model. Horizontal lines indicate 95% confidence intervals, and the vertical black line indicates no effect. Pooled estimates are presented with CR2 robust 95% confidence intervals, whereas 95% prediction intervals are model-based. GRADE indicates the certainty of evidence. CI, confidence interval; PI, prediction interval.

The 95% prediction interval ranged from −0.980 to 0.658, crossed the null value, and encompassed effects favoring both caffeine and placebo. Total heterogeneity was substantial (I^2^_total_ = 60.08%), of which 37.78% was attributable to between-study heterogeneity at Level 3 and 22.30% to heterogeneity among effect sizes within studies at Level 2. Detailed variance components and heterogeneity estimates are provided in **Supplementary Table S9**. Overall, caffeine supplementation did not significantly improve perceptual recovery after exercise.

#### 3.4.2 Effects of caffeine on functional performance during recovery

As shown in **Figure 3**, nine studies involving 125 unique participants contributed 30 effect sizes for functional performance during recovery, including outcomes related to maximum voluntary isometric contraction (MVIC), muscular strength, jumping, sprinting, and endurance performance. The three-level random-effects model indicated that caffeine supplementation produced a small beneficial effect on post-exercise functional performance during recovery, with the pooled effect reaching statistical significance (Hedges’ *g* = −0.208, CR2 95% CI −0.393 to −0.023, CR2 *p* = 0.035).

**Figure 3:**
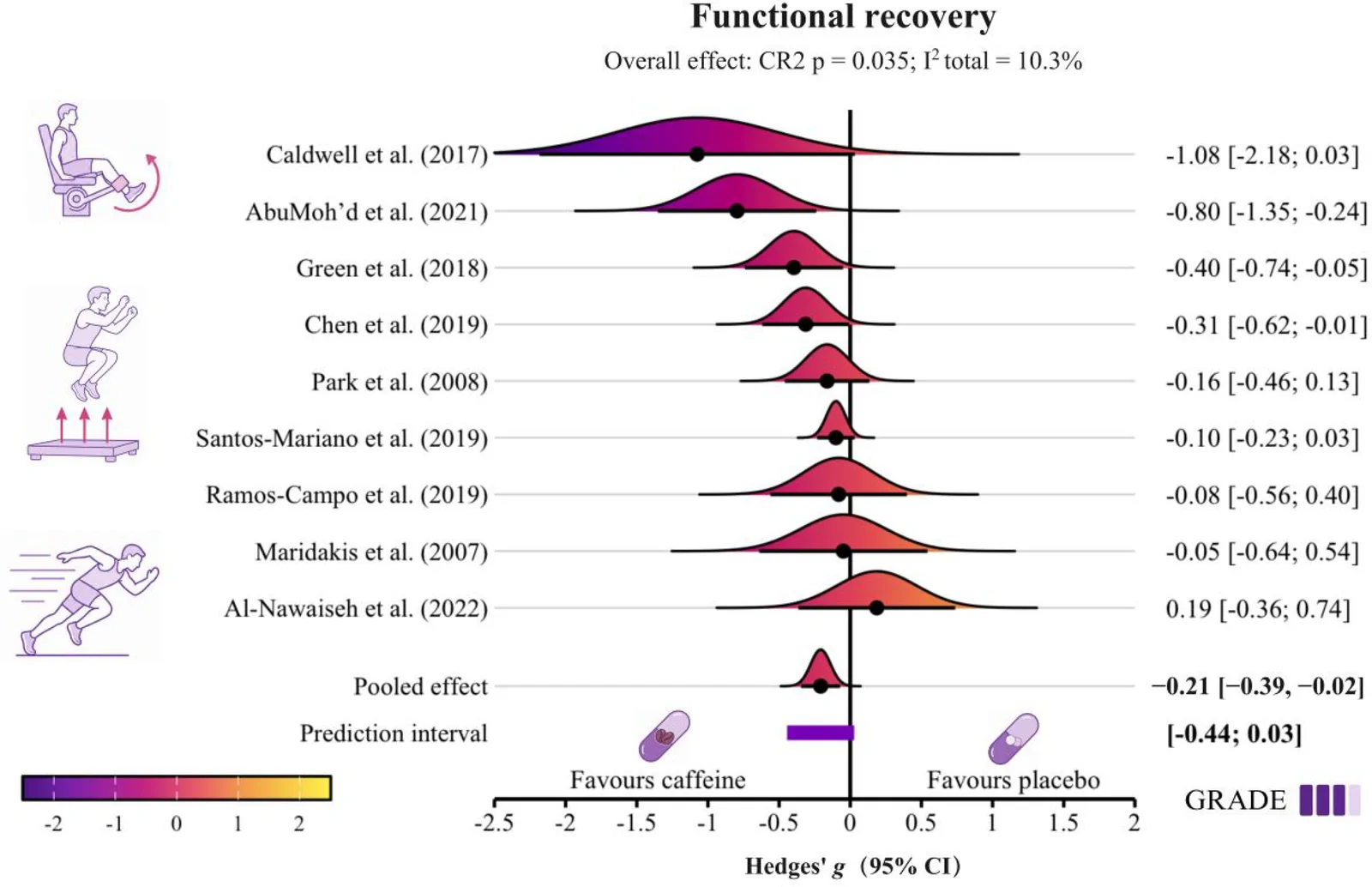
Distribution-enhanced forest plot of the effects of caffeine supplementation on functional performance during recovery. Negative effect sizes favor caffeine, whereas positive values favor placebo. For visualization, multiple effect sizes from the same study within a recovery domain were combined using inverse-variance weighting to obtain a study-level Hedges’*g* and standard error; these summaries were used only for graphical display, whereas pooled estimates were based on all effect-level data in the three-level random-effects model. Horizontal lines indicate 95% confidence intervals, and the vertical black line indicates no effect. Pooled estimates are presented with CR2 robust 95% confidence intervals, whereas 95% prediction intervals are model-based. GRADE indicates the certainty of evidence. CI, confidence interval; PI, prediction interval.

The 95% prediction interval ranged from −0.444 to 0.028, with the upper bound slightly crossing the null value, suggesting that the true effect in future comparable studies may range from a small beneficial effect to little or no effect. Total heterogeneity was low (I^2^_total_ = 10.28%) and was entirely attributable to between-study heterogeneity at Level 3; heterogeneity among effect sizes within studies at Level 2 was 0%. Detailed variance components and heterogeneity estimates are provided in **Supplementary Table S9**. Overall, caffeine supplementation may produce a small improvement in functional performance during recovery; however, this finding should be interpreted cautiously because the prediction interval slightly crossed the null value.

#### 3.4.3 Effects of caffeine on physiological recovery

As shown in **Figure 4**, five studies involving 66 unique participants contributed 36 effect sizes for physiological recovery, including outcomes related to creatine kinase, lactate dehydrogenase, myoglobin, aspartate aminotransferase, alanine aminotransferase, and markers of oxidative stress. The three-level random-effects model showed that the pooled effect slightly favored caffeine but did not reach statistical significance (Hedges’*g* = −0.104, CR2 95% CI −0.346 to 0.138, CR2 *p* = 0.293).

**Figure 4:**
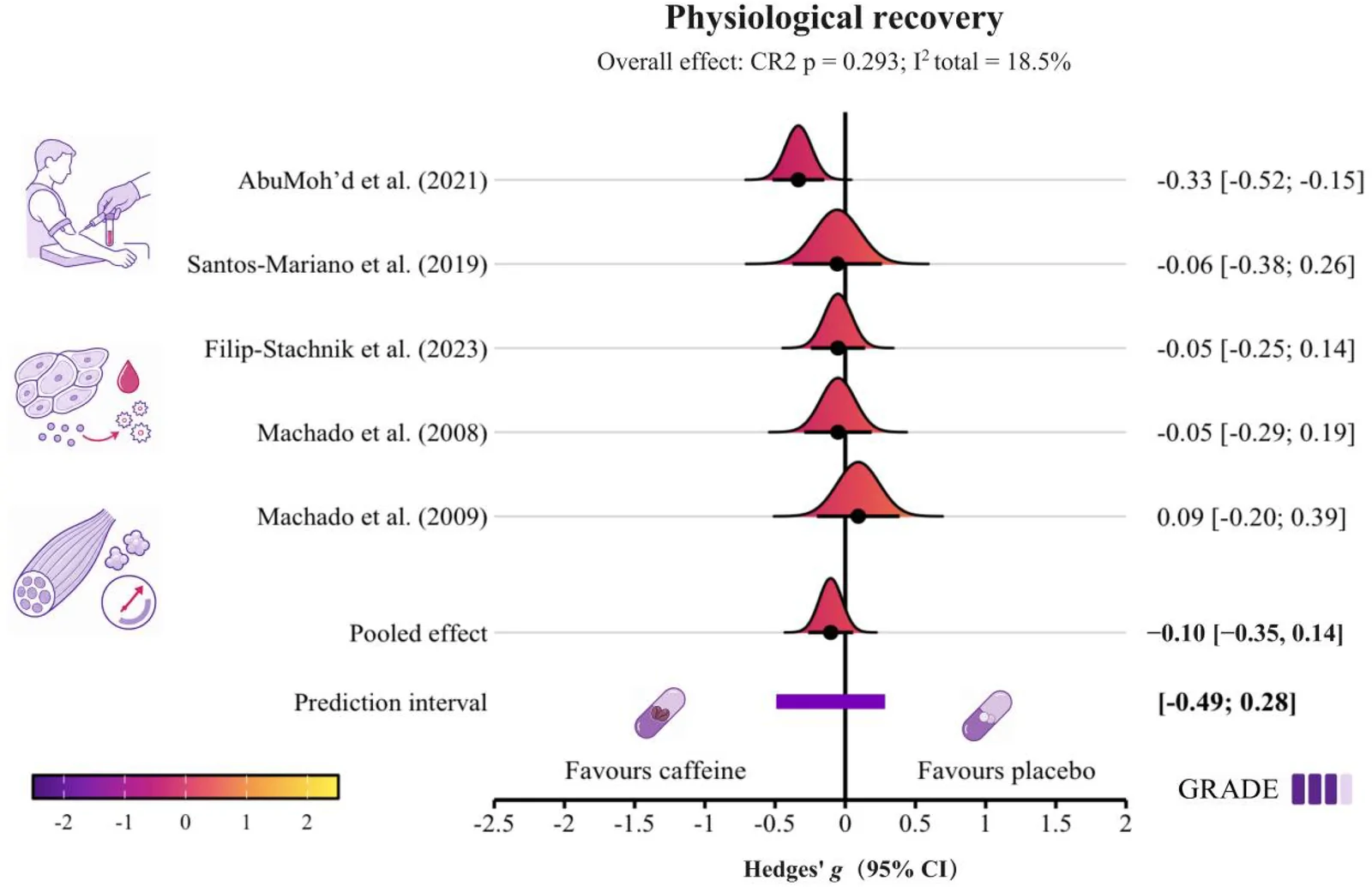
Distribution-enhanced forest plot of the effects of caffeine supplementation on physiological recovery after exercise. Negative effect sizes favor caffeine, whereas positive values favor placebo. For visualization, multiple effect sizes from the same study within a recovery domain were combined using inverse-variance weighting to obtain a study-level Hedges’g and standard error; these summaries were used only for graphical display, whereas pooled estimates were based on all effect-level data in the three-level random-effects model. Horizontal lines indicate 95% confidence intervals, and the vertical black line indicates no effect. Pooled estimates are presented with CR2 robust 95% confidence intervals, whereas 95% prediction intervals are model-based. GRADE indicates the certainty of evidence. CI, confidence interval; PI, prediction interval.

The 95% prediction interval ranged from −0.492 to 0.284, crossed the null value, and encompassed effects in both directions. Total heterogeneity was low (I^2^_total_ = 18.54%), with 8.79% attributable to between-study heterogeneity at Level 3 and 9.74% to heterogeneity among effect sizes within studies at Level 2. Detailed variance components and heterogeneity estimates are provided in **Supplementary Table S9**. Overall, the available evidence did not indicate that caffeine supplementation significantly improved physiological recovery after exercise.

#### 3.4.4 Effects of caffeine on sleep recovery

As shown in **Figure 5**, four studies involving 40 unique participants contributed 28 effect sizes for sleep recovery, including sleep onset latency, sleep efficiency, total sleep time, wake after sleep onset, number of nocturnal awakenings, and subjective sleep quality. The three-level random-effects model showed that caffeine supplementation had a significant detrimental effect on post-exercise sleep recovery (Hedges’*g* = 0.751, CR2 95% CI 0.244 to 1.258, CR2 *p* = 0.018). Because positive effect sizes indicate results favoring placebo, this finding suggests that caffeine may impair sleep recovery after exercise.

**Figure 5:**
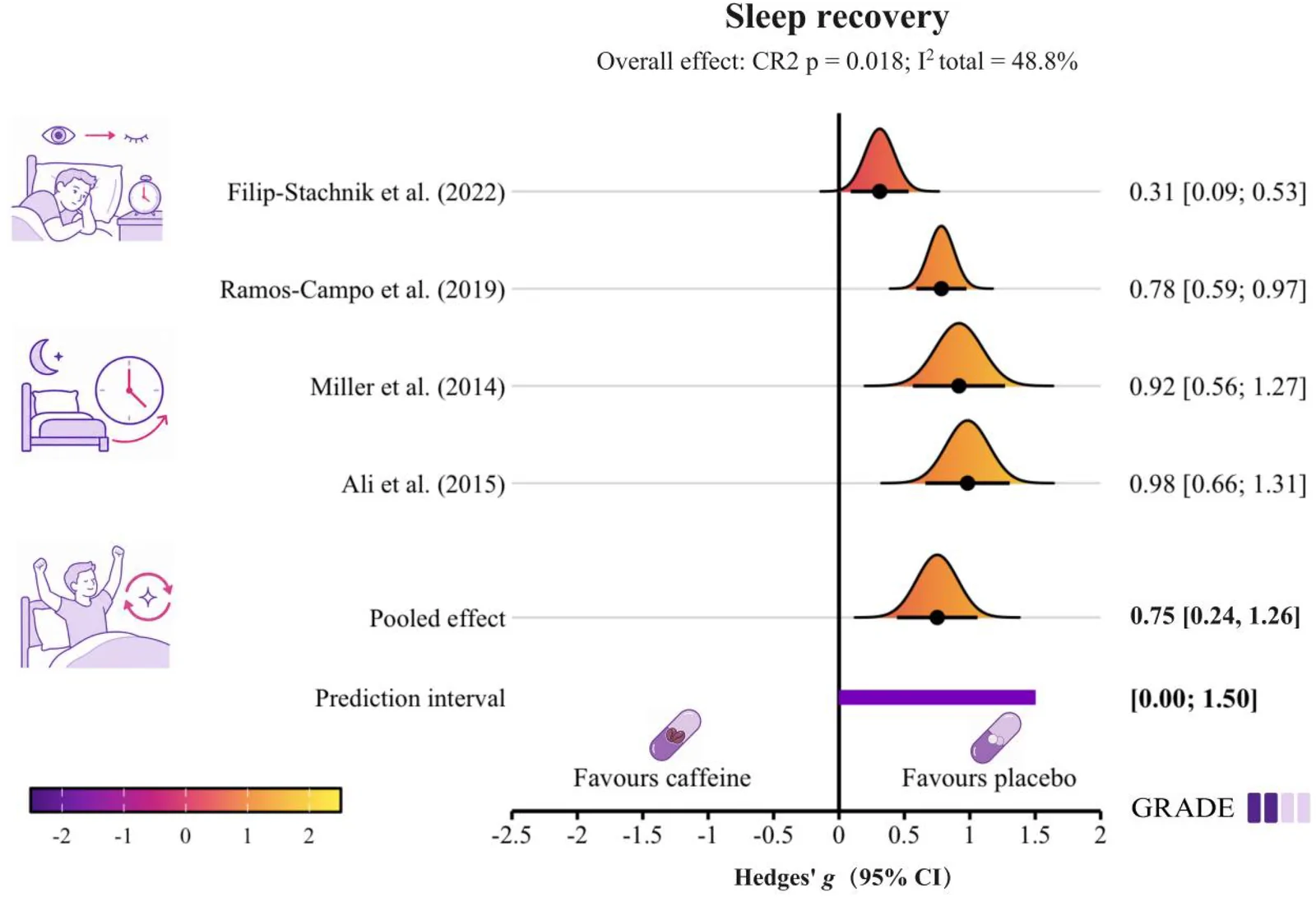
Distribution-enhanced forest plot of the effects of caffeine supplementation on sleep recovery after exercise. Negative effect sizes favor caffeine, whereas positive values favor placebo. For visualization, multiple effect sizes from the same study within a recovery domain were combined using inverse-variance weighting to obtain a study-level Hedges’*g* and standard error; these summaries were used only for graphical display, whereas pooled estimates were based on all effect-level data in the three-level random-effects model. Horizontal lines indicate 95% confidence intervals, and the vertical black line indicates no effect. Pooled estimates are presented with CR2 robust 95% confidence intervals, whereas 95% prediction intervals are model-based. GRADE indicates the certainty of evidence. CI, confidence interval; PI, prediction interval.

The 95% prediction interval ranged from −0.002 to 1.503, with the lower bound close to the null value, suggesting that the true effect in future comparable studies may range from little or no effect to a large detrimental effect. Total heterogeneity was moderate (I^2^_total_ = 48.83%), with 28.50% attributable to between-study heterogeneity at Level 3 and 20.33% to heterogeneity among effect sizes within studies at Level 2. Detailed variance components and heterogeneity estimates are provided in **Supplementary Table S9**. Overall, caffeine supplementation may significantly impair sleep recovery after exercise; however, the findings should be interpreted cautiously because of the small number of included studies.

### 3.5 Exploratory overall pooled effect and moderating effect of recovery domain

To explore the overall effect of caffeine supplementation on post-exercise recovery, the perceptual, functional, physiological, and sleep recovery domains were combined after harmonizing the direction of effect. A total of 120 effect sizes from 16 studies were included. The three-level random-effects model showed that the exploratory overall pooled effect was not statistically significant (Hedges’*g* = 0.044, CR2 95% CI: −0.216 to 0.304, df = 14.62, CR2 *p* = 0.724). The 95% prediction interval ranged from −0.935 to 1.022, and total heterogeneity was substantial (I^2^_total_ = 66.94%). These findings suggest that the direction and magnitude of the effects may differ considerably across recovery domains.

Recovery domain was then examined as a categorical moderator. As shown in **Figure 6**, caffeine produced a small beneficial effect on functional performance during recovery and a clear detrimental effect on sleep recovery, whereas no clear effects were observed for perceptual or physiological recovery. The CR2 robust Wald test indicated a significant moderating effect of recovery domain [*F*(3, 3.88) = 9.76, p = 0.028], demonstrating that the effect in at least one of the four domains differed from those in the other domains. Effects in opposite directions may have offset one another in the overall pooled analysis, resulting in an overall effect close to zero.

**Figure 6:**
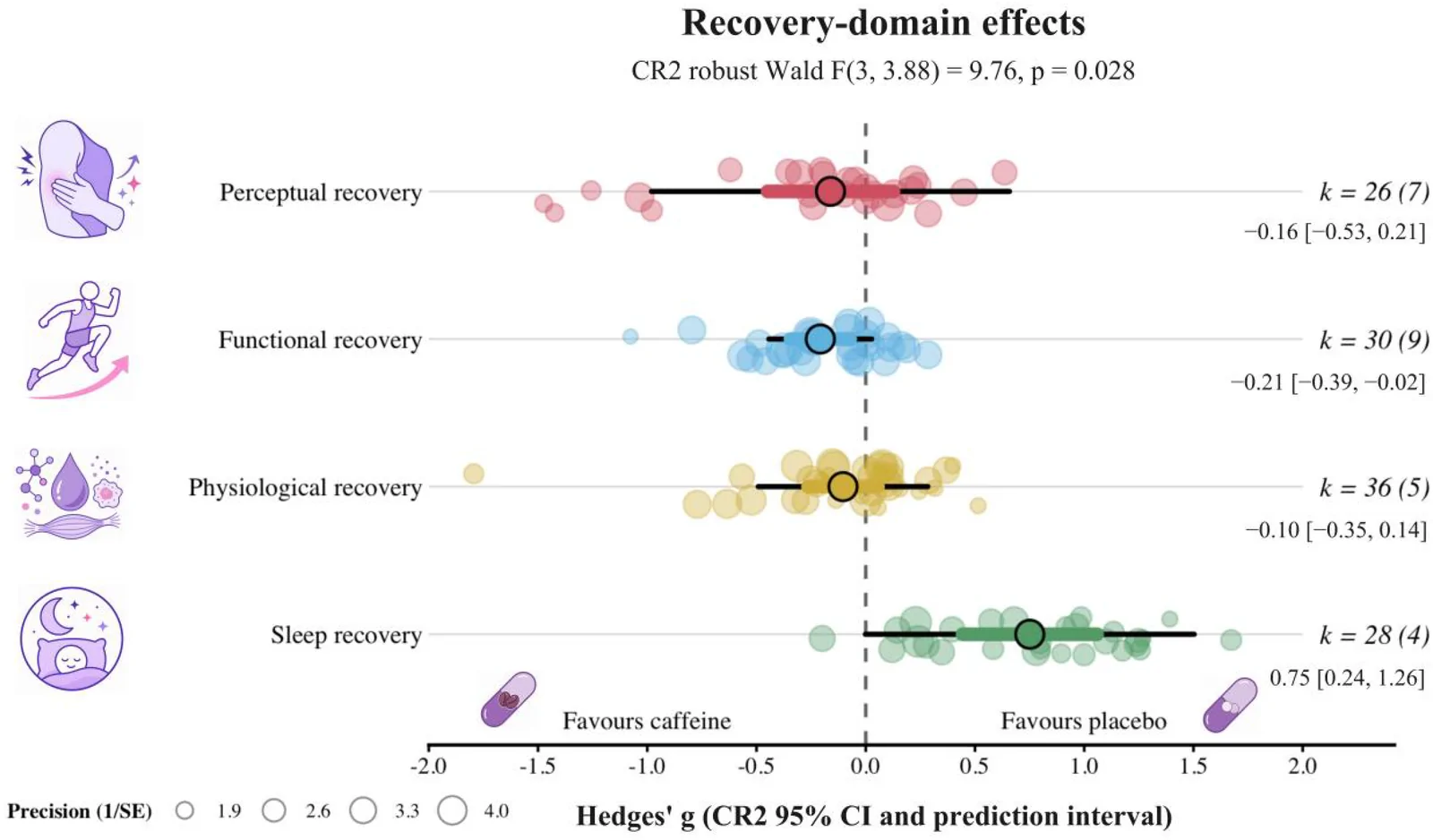
Orchard plot of recovery-domain effects of caffeine supplementation on post-exercise recovery. Semi-transparent circles represent individual effect sizes within each recovery domain, with circle size proportional to precision (1/SE). Large circles indicate the pooled Hedges’*g* for each domain. Colored thick horizontal lines represent CR2 robust 95% confidence intervals, whereas black thin horizontal lines represent model-based 95% prediction intervals from the three-level random-effects models. Negative values favor caffeine, whereas positive values favor placebo. k denotes the number of effect sizes, and the number in parentheses denotes the number of independent studies. Differences across recovery domains were tested using a three-level mixed-effects meta-regression with CR2 robust variance estimation and the HTZ small-sample correction; the omnibus test was CR2 robust Wald F(3, 3.88) = 9.76, p = 0.028.

It should be noted that this omnibus test does not directly indicate that statistically significant differences exist between every pair of recovery domains. Therefore, the overall pooled result should be interpreted as exploratory, and the primary conclusions should continue to be based on the separate models for each recovery domain.

### 3.6 Moderator analyses and meta-regression results

As shown in **Figure 7**, the potential moderating effects of recovery time, caffeine dose, ingestion timing or strategy, and exercise type or recovery model were explored. Because recovery domain significantly moderated the effect estimates, it was included as a covariate in analyses pooling multiple recovery domains. Sleep outcomes were excluded from the recovery-time analysis, which included 92 effect sizes from 13 studies; the remaining moderator analyses included 120 effect sizes from 16 studies.

**Figure 7:**
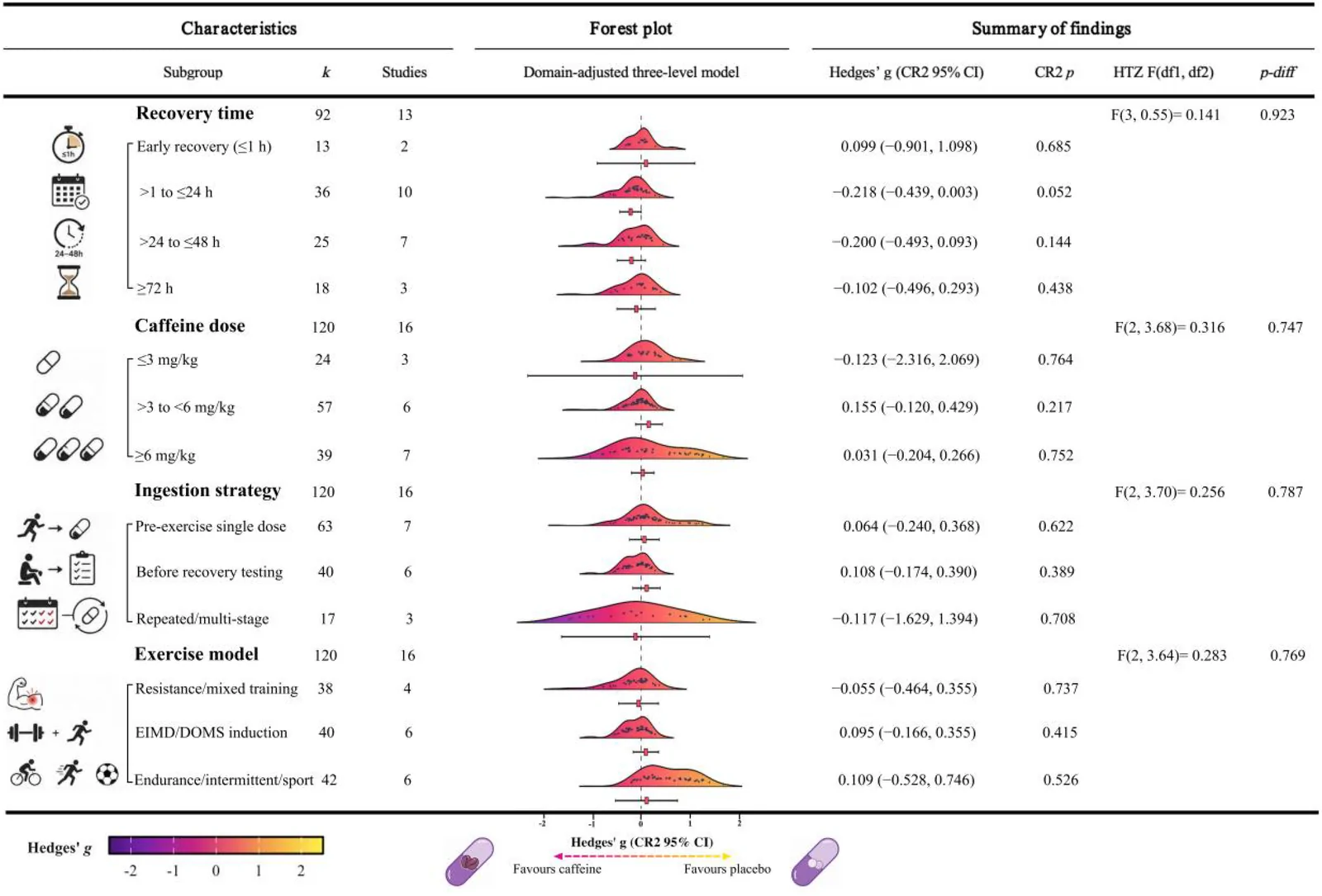
Domain-adjusted moderator analyses of the effects of caffeine supplementation on post-exercise recovery. Moderator analyses were conducted using three-level mixed-effects meta-regression models adjusted for recovery domain. Subgroup estimates and 95% confidence intervals were based on CR2 robust inference with Satterthwaite small-sample correction, and overall moderator effects were tested using CR2 robust HTZ Wald *F* tests. Negative Hedges’*g* values favor caffeine, whereas positive values favor placebo. k, number of effect sizes; Studies, number of independent studies; CR2 *p*, robust *p* value for the subgroup estimate; *p*-diff, *p* value for the overall moderator test. EIMD, exercise-induced muscle damage; DOMS, delayed-onset muscle soreness.

After adjustment for recovery domain, the overall difference across recovery-time categories was not statistically significant [*F*(3, 0.55) = 0.141, *p* = 0.923]. Adjusted estimates were close to the null during early recovery (≤1 h; *g* = 0.099), whereas estimates beyond 1 h generally favored caffeine (*g* = −0.218 to −0.102). However, the very limited effective degrees of freedom and wide confidence intervals substantially constrained inference, and the available evidence was insufficient to establish a consistent moderating effect of recovery time.

No significant overall difference was observed across caffeine-dose categories [*F*(2, 3.68) = 0.316, *p* = 0.747]. The adjusted estimates were small and imprecise across ≤3, >3 to<6, and ≥6 mg/kg dose categories, providing no clear evidence of a dose-dependent pattern. Similarly, ingestion timing or strategy did not significantly moderate the effect of caffeine [*F*(2, 3.70) = 0.256, *p* = 0.787], with estimates varying only modestly across pre-exercise, pre-recovery-test, and repeated or multi-stage ingestion strategies.

Exercise type or recovery model was also not a significant moderator after adjustment for recovery domain [*F*(2, 3.64) = 0.283, *p* = 0.769]. Adjusted estimates were close to the null across conventional resistance or mixed training, EIMD/DOMS induction models, and endurance, intermittent, or sport-specific exercise. Given the small number of independent studies in several categories and the resulting imprecision, these moderator findings should be regarded as exploratory and inconclusive rather than evidence that moderation is absent. Detailed domain-adjusted estimates are presented in **Figure 7**.

A further domain-adjusted meta-regression examined relative caffeine dose as a continuous variable (**Figure 8**). No clear linear association was observed between caffeine dose and post-exercise recovery (*β* = 0.048, CR2 95% CI −0.209 to 0.306, *df* = 3.545, *p* = 0.615). Given the limited number of independent studies, relatively narrow dose range, and imprecision of the estimate, this analysis should be considered exploratory and does not provide sufficient evidence for a stable dose–response relationship.

**Figure 8:**
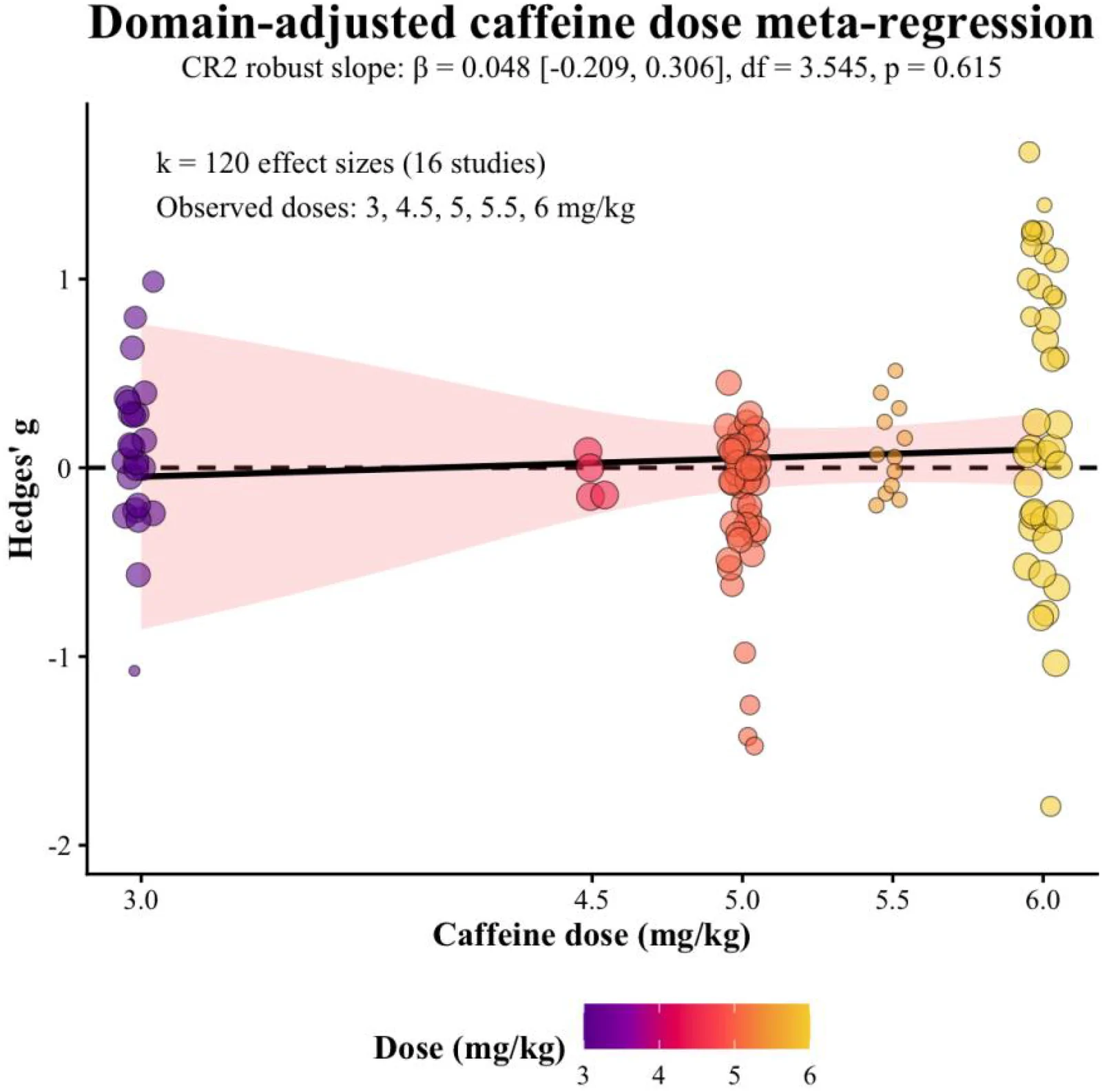
Domain-adjusted meta-regression of the association between caffeine dose and post-exercise recovery. The association between relative caffeine dose and post-exercise recovery was examined using a three-level mixed-effects meta-regression adjusted for recovery domain. The solid black line represents the fitted regression line and the shaded area the CR2 robust 95% confidence interval. Circle size is proportional to precision, and color indicates caffeine dose. The dashed horizontal line denotes the null effect (Hedges’*g* = 0). Negative values favor caffeine, whereas positive values favor placebo.

### 3.7 Sensitivity analyses

To assess the robustness of the primary findings, sensitivity analyses were conducted using alternative within-participant correlation coefficients for crossover trials, leave-one-study-out analyses, domain-specific exclusion of studies judged to be at high risk of bias, a one-study-one-domain analysis, and additional analyses addressing potential acute or residual ergogenic effects on functional performance. Detailed results are presented in **Supplementary Table S4**.

Across alternative within-participant correlation assumptions (*r* = 0.30, 0.50, and 0.70), the direction and statistical conclusions for perceptual, physiological, and sleep recovery remained unchanged. Functional performance consistently favored caffeine, although statistical significance was not retained under the *r* = 0.30 assumption (CR2 *p* = 0.060). Leave-one-study-out analyses likewise showed stable effect directions across all four domains, although the statistical significance of the functional and sleep findings varied after exclusion of individual studies.

Excluding studies judged to be at high risk of bias within each recovery domain did not materially alter the findings. Perceptual recovery remained non-significant after exclusion of Filip-Stachnik et al. (2023); Maridakis et al. (2007), and Hurley et al. (2013) (Hedges’*g* = −0.159, CR2 *p* = 0.603). Functional performance remained favorable to caffeine after exclusion of Caldwell et al. (2017) and Maridakis et al. (2007) (*g* = −0.203, CR2 *p* = 0.049), whereas physiological recovery remained non-significant after exclusion of Filip-Stachnik et al. (2023) (*g* = −0.117, CR2 *p* = 0.390). No high-risk study contributed to the sleep domain.

In the one-study-one-domain sensitivity analysis, each study contributed only one composite effect within each recovery domain. The resulting estimates were similar to the primary analyses for perceptual (*g* = −0.198, CR2 *p* = 0.312), functional (*g* = −0.248, CR2 *p* = 0.032), physiological (*g* = −0.143, CR2 *p* = 0.226), and sleep outcomes (*g* = 0.782, CR2 *p* = 0.024). Thus, reducing densely reported studies to a single study-level effect per domain did not materially alter the direction, magnitude, or statistical interpretation of the pooled estimates.

Additional sensitivity analyses examined whether the functional finding could reflect acute or residual ergogenic effects. After excluding assessments performed within 1 h after exercise, the effect remained favorable to caffeine but was attenuated and no longer statistically significant (*g* = −0.153, CR2 95% CI −0.353 to 0.048, CR2 *p* = 0.089). Excluding outcomes preceded by caffeine ingestion specifically before recovery testing left only three studies, and applying both restrictions simultaneously left only two studies; estimates were consequently highly imprecise. These analyses therefore did not provide sufficient evidence to determine whether the observed functional improvement reflected accelerated recovery rather than acute or residual ergogenic effects.

Overall, effect directions were generally stable across sensitivity analyses. Perceptual and physiological findings were comparatively robust, whereas functional and sleep findings showed greater sensitivity to analytical assumptions or individual studies.

### 3.8 Publication bias and small-study effects

To assess potential small-study effects and funnel-plot asymmetry, a domain-adjusted three-level Egger regression was first performed using the overall dataset, with inference based on CR2 robust variance estimation and study as the clustering unit. The regression coefficient for the standard error of the effect size was not statistically significant (β = −0.670, CR2 95% CI: −5.748 to 4.408, CR2 *p* = 0.753). Thus, after accounting for differences across recovery domains and the dependence of effect sizes within studies, no clear evidence of small-study effects or funnel-plot asymmetry was observed. For descriptive visualization, contour-enhanced funnel plots were additionally constructed using one study-level composite effect for each study within each recovery domain, thereby avoiding visual overrepresentation of studies contributing multiple outcomes or assessment time points (**Figure 9**). These plots were used for visual assessment only and did not replace the effect-level three-level Egger analyses.

**Figure 9:**
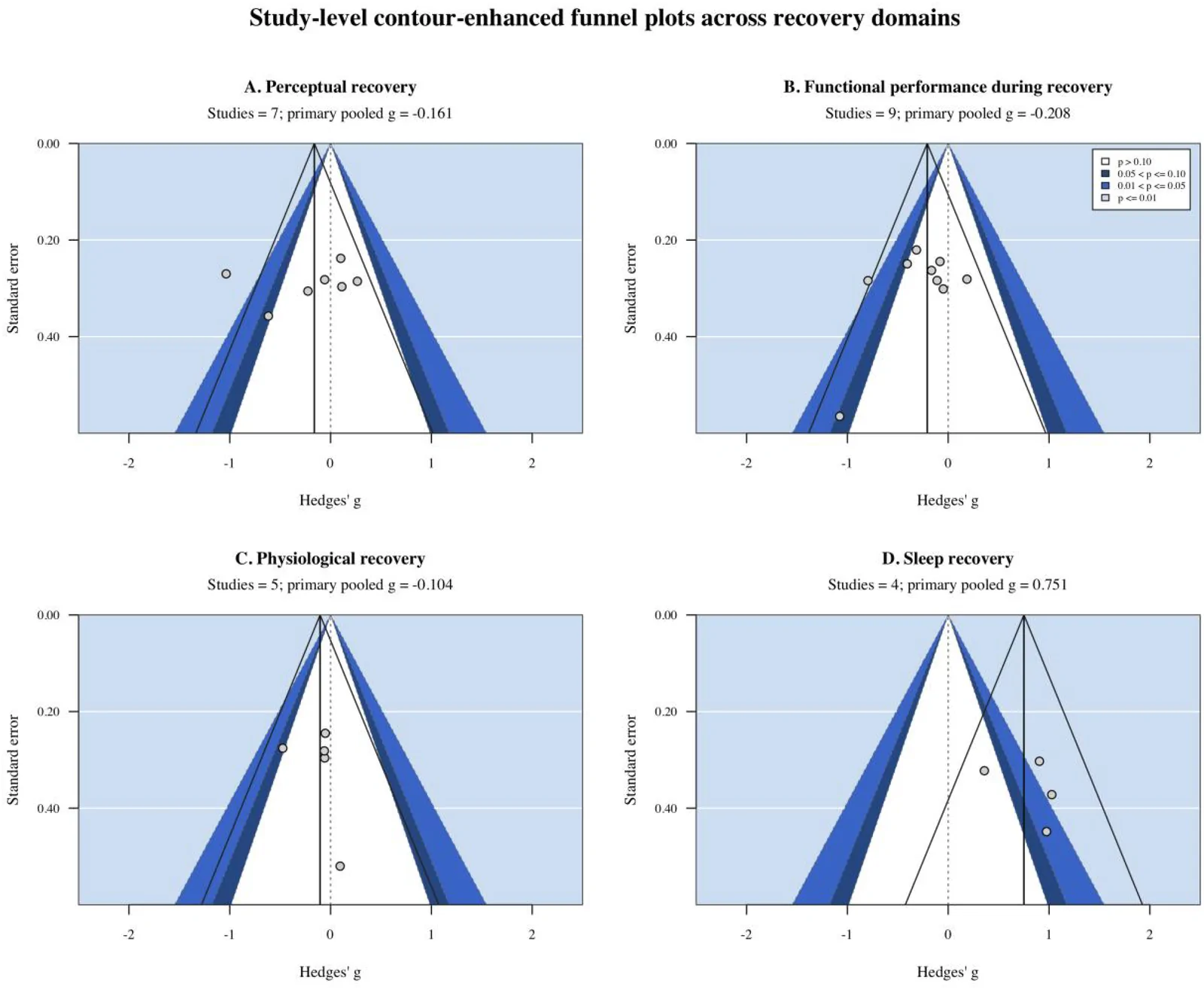
Study-level contour-enhanced funnel plots across recovery domains. Panels **(A–D)** represent perceptual recovery, functional performance during recovery, physiological recovery, and sleep recovery, respectively. Each circle represents one study-level composite effect. Background shading indicates two-sided statistical-significance contours; solid vertical lines indicate domain-specific pooled effects, and dashed vertical lines indicate the null effect (Hedges’*g* = 0). Because fewer than 10 independent studies contributed to each domain, the plots should be interpreted descriptively.

Exploratory three-level Egger regressions were then conducted separately for the four recovery domains. The tests were not statistically significant for perceptual recovery, functional performance during recovery, or physiological recovery, with CR2 *p* values of 0.297, 0.155, and 0.182, respectively. In the sleep recovery domain, however, the regression coefficient was statistically significant (*β* = 8.488, CR2 95% CI: 2.007 to 14.970, CR2 *p* = 0.029), suggesting possible small-study effects or funnel-plot asymmetry. Nevertheless, fewer than 10 independent studies were included in each domain, and the sleep recovery domain comprised only four studies. Therefore, the domain-specific findings should be interpreted as exploratory and cannot be regarded as definitive evidence regarding the presence or absence of small-study effects (**Supplementary Table S5**; **Supplementary Figure S1A**).

The power-enhanced funnel plots indicated that, assuming the true effect was equal to the pooled effect estimated by the corresponding domain-specific three-level model, most effect sizes in the perceptual, functional, and physiological recovery domains were located in regions of relatively low theoretical statistical power, whereas theoretical power was comparatively higher in the sleep recovery domain. This analysis was used primarily to describe theoretical statistical power across different levels of standard error and the distribution of effect sizes with low power; it was not used as an independent criterion for determining publication bias (**Supplementary Figure S1B**).

### 3.9 Certainty of evidence

The GRADE assessment indicated that the certainty of evidence was very low for perceptual recovery, moderate for functional and physiological recovery, and low for sleep recovery. For perceptual recovery, the certainty of evidence was downgraded by one level each for risk of bias, inconsistency, and imprecision. Functional and physiological recovery were each downgraded by one level for imprecision, whereas no downgrading was applied for risk of bias, inconsistency, or indirectness in these domains.

For sleep recovery, the certainty of evidence was downgraded by one level for risk of bias and one level for imprecision. All four studies contributing to this domain were rated as having some concerns in the study-by-recovery-domain RoB 2 assessment. Although the pooled effect indicated poorer sleep recovery with caffeine (Hedges’*g* = 0.751, CR2 95% CI 0.244 to 1.258), only four independent studies involving 40 unique participants contributed to this analysis, and the model-based 95% prediction interval extended from −0.002 to 1.503. Together with some sensitivity of statistical significance to alternative analytical assumptions and individual studies, these findings resulted in downgrading for imprecision.

No recovery domain was downgraded for indirectness. Publication bias could not be reliably assessed because fewer than 10 independent studies contributed to each recovery domain. Non-significant exploratory Egger tests were therefore not interpreted as evidence that publication bias was absent. Although the exploratory Egger regression for sleep recovery suggested possible small-study effects, the estimate was based on only four independent studies and was considered unstable. Accordingly, no additional downgrading for publication bias was applied. Detailed evidence profiles and reasons for downgrading are presented in **Supplementary Table S6**.

## 4 Discussion

### 4.1 Main findings

Within a unified multidimensional recovery framework, this study used three-level meta-analysis to evaluate the effects of caffeine supplementation on perceptual, functional, physiological, and sleep recovery after exercise. The findings demonstrated clear domain specificity in the effects of caffeine. Caffeine may produce a small improvement in functional performance during the recovery period, but it did not clearly improve perceptual or physiological recovery and may impair subsequent sleep recovery. The overall moderating effect of recovery domain was statistically significant, indicating that the direction and magnitude of the effects differed across recovery dimensions. The exploratory overall pooled effect was close to zero, which may largely reflect the offsetting of potentially beneficial functional effects by adverse effects on sleep and should not be interpreted simply as evidence that caffeine has no effect on post-exercise recovery.

The certainty of evidence was moderate for functional and physiological recovery, low for sleep recovery, and very low for perceptual recovery. These certainty ratings reflect confidence in the estimated effects rather than the magnitude or practical importance of those effects; therefore, moderate-certainty evidence should not be interpreted as indicating a substantial practical benefit. Across sensitivity analyses, the direction of the pooled effect within each domain remained generally stable; however, the statistical significance of the functional and sleep findings varied under alternative correlation assumptions or after individual studies were excluded. In particular, although the average sleep effect indicated poorer recovery with caffeine, only four independent studies contributed to this domain, the CR2 confidence interval was relatively wide, and the model-based prediction interval extended to approximately the null. Accordingly, the adverse sleep finding should be interpreted cautiously, particularly with respect to its magnitude and consistency across settings.

Taken together, the current evidence does not support viewing caffeine as a supplement that comprehensively enhances post-exercise recovery. Its potential value appears to depend on the specific recovery dimension and practical context. Any short-term maintenance or enhancement of functional performance during recovery should therefore be weighed against the possibility of impaired subsequent sleep, while recognizing the remaining uncertainty and the limited certainty of evidence in some recovery domains.

### 4.2 Potential effects of caffeine on functional performance during recovery

The present study found that caffeine may produce a small beneficial effect on functional performance during the post-exercise recovery period, with moderate-certainty evidence for this domain. Although the primary pooled estimate reached statistical significance, the magnitude of the effect was small, and this statistical finding should not be interpreted as evidence of a substantial or practically meaningful acceleration of recovery. This finding is broadly consistent with previous evidence regarding the acute ergogenic effects of caffeine. A previous meta-analysis showed that caffeine produced small improvements in muscular strength (SMD = 0.20, 95% CI: 0.03 to 0.36) and power performance (SMD = 0.17, 95% CI: 0.00 to 0.34) (Grgic et al., 2018). The position stand of the International Society of Sports Nutrition similarly concluded that caffeine can improve muscular strength and endurance, movement velocity, jumping, sprinting, and both aerobic and anaerobic exercise performance (Guest et al., 2021). However, previous reviews have primarily focused on immediate ergogenic effects during exercise, whereas the present study evaluated functional performance during the post-exercise recovery period. Therefore, the observed beneficial effect may be more appropriately interpreted as evidence that caffeine helps individuals temporarily maintain or enhance exercise capacity while fatigued or experiencing muscle damage.

This effect may be mediated primarily through antagonism of adenosine receptors, attenuation of central inhibition, and reductions in perceived fatigue, pain, and exertion. Following ingestion of 6 mg/kg caffeine, isokinetic peak torque increased by 6.8% in non-damaged muscle and by 9.4% in damaged muscle, whereas maximum voluntary isometric contraction, fatigue index, and muscle soreness did not improve (Green et al., 2018). Similarly, caffeine improved maximum voluntary isometric contraction following exercise-induced muscle damage, although the magnitude of this effect may have been influenced by sex and differences in pain responses (Chen et al., 2019). In addition, caffeine increased countermovement-jump height and power at 48 and 72 h after eccentric exercise but did not improve maximum voluntary contraction, peripheral fatigue, or sprint performance (Santos-Mariano et al., 2019). These findings suggest that caffeine may preferentially improve certain measures of dynamic strength or explosive performance rather than producing consistent benefits across all functional outcomes.

Importantly, improved functional performance during recovery should not be equated with accelerated muscle-tissue repair or overall recovery. Previous research showed that caffeine increased maximum voluntary isometric contraction in non-damaged muscle by 10.4% but had no clear effect on strength or muscle activation in damaged muscle, suggesting that muscle damage may constrain its ergogenic effects (Park et al., 2008). A systematic review likewise indicated that caffeine may reduce pain perception or improve selected functional outcomes, but these effects were not consistently accompanied by reductions in biomarkers of muscle damage and therefore should not be interpreted as evidence that exercise-induced muscle damage itself was alleviated (Caldas et al., 2022). Furthermore, studies involving pre-exercise caffeine ingestion are susceptible to residual acute ergogenic effects, making it difficult to distinguish between “accelerated recovery” and a temporary improvement in performance before full recovery has occurred (Matsumura et al., 2026). Given the absence of a clear improvement in physiological recovery in the present study, a more plausible interpretation is that caffeine may temporarily maintain or enhance functional output during the recovery period, whereas current evidence remains insufficient to conclude that it promotes muscle-tissue repair or shortens the overall recovery process.

### 4.3 Effects of caffeine on perceptual and physiological recovery

The present study found no clear overall benefit of caffeine on post-exercise perceptual recovery, and the certainty of evidence for this domain was very low. Previous findings have likewise been inconsistent. One meta-analysis reported that caffeine reduced muscle soreness at 48 h after exercise, whereas no significant differences were observed at other time points (Muljadi et al., 2021). After further distinguishing between pre- and post-exercise ingestion, 5–6 mg/kg caffeine did not significantly reduce DOMS-related pain at 24, 48, or 72 h (Atalay et al., 2025). Another systematic review suggested that repeated caffeine ingestion between 24 and 72 h after muscle-damaging exercise may reduce pain perception by 3.9%–26%, whereas the effects of a single dose were inconsistent (Caldas et al., 2022). In young elite football players, ingestion of 400 mg caffeine also failed to significantly improve delayed-onset muscle soreness, rating of perceived exertion, or heart-rate recovery at 24 h post-exercise (Bezuglov et al., 2025). The inconsistent findings across studies may reflect differences in ingestion strategy, exercise model, assessment timing, and individual characteristics.

Caffeine may reduce pain transmission and perception through antagonism of central and peripheral adenosine receptors, but this effect does not appear to be consistent. Ingestion of 5 mg/kg caffeine after exercise-induced muscle damage reduced pain intensity during maximum voluntary isometric contraction by 48% (Maridakis et al., 2007) and may also reduce muscle soreness on the second and third days after exercise (Hurley et al., 2013). However, other studies have not reproduced these findings, suggesting that the analgesic effects of caffeine may be time- and individual-dependent. Because pain and soreness are subjective outcomes, they are readily influenced by expectancy effects, habitual caffeine consumption, sex, and the initial severity of muscle damage. Accordingly, a transient reduction in pain should not be interpreted directly as evidence of accelerated recovery.

The present study also found no clear improvement in creatine kinase, lactate dehydrogenase, myoglobin, or other physiological recovery markers following caffeine supplementation, with moderate-certainty evidence for the physiological recovery domain. Previous systematic evidence indicates that a single dose of caffeine ingested before or after exercise generally does not reduce circulating creatine kinase concentrations, and that changes in pain perception are not consistently associated with changes in biomarkers of muscle damage (Caldas et al., 2022). Following ingestion of 3 mg/kg caffeine, no significant changes were observed in creatine kinase, lactate dehydrogenase, or most markers of oxidative stress (Filip-Stachnik et al., 2023). Similarly, in professional football players, ingestion of 5.5 mg/kg caffeine did not alter creatine kinase, lactate dehydrogenase, aspartate aminotransferase, or alanine aminotransferase concentrations between 24 and 72 h after exercise (MaChado et al., 2009). It should be noted that blood biomarkers such as creatine kinase show substantial inter-individual variability and are influenced by sampling time, training status, and the repeated-bout effect. Previous studies may also have relied excessively on indirect markers such as creatine kinase, while remaining unable to distinguish the acute ergogenic effects of pre-exercise caffeine from genuine recovery from muscle damage (Matsumura et al., 2026). Overall, caffeine may reduce subjective discomfort under certain conditions, but current evidence is insufficient to conclude that it attenuates muscle damage or accelerates physiological repair. These findings further indicate that perceptual, functional, and physiological recovery may not occur in parallel.

### 4.4 Potential adverse effects of caffeine on sleep recovery

The present meta-analysis indicated that caffeine supplementation was associated with poorer post-exercise sleep recovery on average (Hedges’*g* = 0.751, CR2 95% CI 0.244 to 1.258), although the certainty of evidence for this domain was low. Only four independent studies involving 40 unique participants contributed to the quantitative synthesis, and the model-based 95% prediction interval extended from −0.002 to 1.503. Thus, although the overall pooled effect was consistent with a potentially adverse effect of caffeine on sleep recovery, uncertainty remains regarding the magnitude and consistency of this effect across different research settings, and the finding should therefore be interpreted cautiously.

Nevertheless, the direction of the pooled estimate was broadly consistent with previous systematic reviews and meta-analyses. Caffeine has commonly been reported to prolong sleep onset latency, reduce total sleep time and sleep efficiency, and increase nocturnal awakenings (Clark and Landolt, 2017). A previous meta-analysis showed that caffeine reduced total sleep time by an average of 45 min and sleep efficiency by 7 percentage points, while increasing sleep onset latency and wake after sleep onset by 9 and 12 min, respectively (Gardiner et al., 2023). A more recent meta-analysis similarly found that caffeine reduced total sleep time by 34.67 min and sleep efficiency by 4.74 percentage points, prolonged sleep onset latency by 8.35 min, and reduced the proportion of slow-wave sleep (Chang et al., 2025).

Evidence specifically involving athletes is also broadly consistent with this pattern. A systematic review of nine randomized controlled trials indicated that caffeine may impair sleep duration, sleep efficiency, sleep initiation, and subjective sleep quality in athletes, although findings for objective sleep measures were not entirely consistent across studies (Bodur et al., 2025). Another meta-analysis found that caffeine ingestion before evening training or competition reduced sleep efficiency by an average of 4.87 percentage points and total sleep time by approximately 32 min, while athletes commonly reported marked subjective sleep impairment (Kocak et al., 2025). However, because relatively few studies were included and most participants were men, these findings should also be interpreted cautiously.

Individual studies further illustrate the potential trade-off between immediate performance benefits and subsequent sleep impairment. Although a total caffeine dose of 6 mg/kg ingested in the evening improved cycling time-trial performance, it prolonged sleep onset latency from 10.2 to 51.1 min, reduced sleep efficiency from 91.5% to 76.1%, and decreased total sleep time from 464 to 391 min (Miller et al., 2014). In another study, 6 mg/kg caffeine did not improve evening 800-m running performance but increased nocturnal awakenings and wakefulness and reduced subjective sleep quality (Ramos-Campo et al., 2019). Female participants also experienced impaired subsequent sleep quality after caffeine ingestion during evening exercise (Ali et al., 2015).

Lower caffeine doses do not necessarily eliminate the possibility of sleep disruption. Although ingestion of 3 mg/kg caffeine before evening training did not significantly alter actigraphy-derived sleep measures or recovery–stress status on the following day, athletes nevertheless reported reduced subjective sleep quality (Filip-Stachnik, 2022). Furthermore, evening ingestion of 6 and 9 mg/kg caffeine improved rowing-ergometer performance but markedly reduced subjective sleep quality and increased next-day sleepiness; by comparison, sleep disruption was less pronounced with 3 mg/kg caffeine (Karakulak et al., 2025). These findings suggest that the effects of caffeine on sleep may be dose-dependent to some extent, although lower doses should not be assumed to be entirely free of sleep-related effects.

These effects may be primarily attributable to caffeine-mediated antagonism of adenosine receptors. The resulting central stimulatory effects may persist until bedtime and may be jointly influenced by caffeine dose, the interval between ingestion and bedtime, habitual caffeine consumption, and individual differences in caffeine metabolism (Clark and Landolt, 2017; Silva et al., 2025). Accordingly, caffeine use in the late afternoon or evening may provide short-term performance benefits while potentially compromising subsequent sleep.

Given the small number of independent studies included in the present sleep analysis, the relatively wide CR2 confidence interval, the prediction interval extending to approximately the null, and the sensitivity of statistical significance in some sensitivity analyses, additional high-quality studies are needed to clarify the magnitude and consistency of this association. For training or competition in the late afternoon or evening, caffeine use should therefore be considered in relation to dose, caffeine ingestion timing, individual sensitivity, and the training or competition schedule on the following day.

### 4.5 Moderating factors, heterogeneity, and robustness of the findings

The exploratory moderator analyses provided no clear evidence that recovery time, caffeine dose, caffeine ingestion timing, or exercise model modified the effects of caffeine on post-exercise recovery after adjustment for recovery domain. For recovery time, the point estimate for the >1–≤24 h category favored caffeine, but its CR2 95% confidence interval extended to approximately the null, and the omnibus test provided no evidence of differences across recovery-time categories. Therefore, the >1–≤24 h period should not be interpreted as a superior recovery window.

Neither the categorical dose analysis nor the continuous-dose meta-regression provided evidence of a dose–response relationship. However, caffeine doses in the available studies were concentrated primarily within the range of approximately 3–6 mg/kg, limiting the ability to characterize effects at lower or higher doses or to identify potential non-linear relationships. Moreover, individual responses to caffeine may vary according to habitual caffeine intake, metabolic characteristics, and genetic variation (Guest et al., 2021; Pickering and Grgic, 2019). However, habitual caffeine consumption was inconsistently reported across the included studies, precluding a formal moderator analysis; therefore, differences in habitual caffeine exposure remain a potential source of between-study heterogeneity. Accordingly, the present evidence does not allow an optimal caffeine dose for post-exercise recovery to be identified.

No clear moderating effect of ingestion timing or strategies was observed after adjustment for recovery domain. Nevertheless, the timing categories are not necessarily physiologically equivalent. Pre-exercise ingestion may primarily reflect caffeine exposure during the exercise bout and its residual effects during subsequent recovery, whereas ingestion after exercise or immediately before a recovery assessment may more directly influence performance, perceived exertion, or pain at the time of outcome measurement. These distinctions are particularly relevant when interpreting functional performance outcomes during recovery because an apparent improvement in post-exercise performance may reflect an acute or residual ergogenic effect rather than accelerated recovery itself (Matsumura et al., 2026).

Similarly, no clear moderating effect of exercise model was observed after adjustment for recovery domain. Because different exercise models contributed different combinations of recovery outcomes, any apparent differences across exercise models should be interpreted cautiously and may partly reflect differences in outcome composition rather than an independent effect of exercise type. Accordingly, the available evidence does not support stratified recommendations according to exercise model, caffeine dose, caffeine ingestion timing, or recovery time.

Because many combinations of recovery domain and moderator category were represented by only one or very few independent studies, reliable domain-by-moderator interaction analyses were not considered feasible. The moderator findings should therefore be regarded as exploratory and inconclusive rather than as evidence that these factors have no moderating effects. Additional studies with more balanced coverage of recovery domains, caffeine doses, ingestion strategies, exercise models, and recovery time points are needed before robust domain-specific moderators can be identified.

The sensitivity analyses indicated that the direction of the pooled effect within each recovery domain remained generally stable. However, statistical significance for functional performance during recovery varied under alternative assumptions regarding the within-participant correlation in crossover trials and after exclusion of individual studies, while the sleep recovery finding was also sensitive to the influence of individual studies. In contrast, conclusions for perceptual and physiological recovery were comparatively stable across the sensitivity analyses. These findings suggest that the primary conclusions are more robust with respect to effect direction than to statistical significance or the precise magnitude of the pooled effects in some domains.

The domain-adjusted overall Egger regression did not identify a statistically significant small-study effect. However, because fewer than 10 independent studies contributed to each recovery domain, non-significant Egger tests cannot reliably exclude publication bias. The exploratory sleep-domain Egger regression suggested possible small-study effects, but this result was based on only four independent studies and should therefore be interpreted cautiously. Taken together, uncertainty remains regarding both the precision of some domain-specific estimates and the possibility of small-study effects. Future studies should use clearly defined and comparable protocols for caffeine dose, caffeine ingestion timing, exercise models, and recovery-outcome assessment (Pickering and Grgic, 2019; Silva et al., 2025).

### 4.6 Practical implications

The findings of the present study indicate that caffeine should not be regarded as a supplement that comprehensively enhances post-exercise recovery. For athletes who need to maintain selected aspects of functional performance over short recovery intervals, caffeine may have some short-term practical value. However, these functional benefits are more likely to reflect a temporary ergogenic effect than a reduction in muscle damage or an acceleration of physiological repair. Accordingly, improvements in performance during the recovery period should not be interpreted as evidence that an athlete has fully recovered, and caffeine should not be considered a primary strategy for relieving muscle soreness or promoting tissue repair.

Caffeine use requires particular consideration of the trade-off between immediate performance benefits and subsequent sleep impairment. Existing position statements generally recommend 3–6 mg/kg caffeine before exercise to enhance performance (Guest et al., 2021), but the present study did not identify an optimal dose, caffeine ingestion timing, or exercise type for promoting recovery. During training or competition in the late afternoon or evening, caffeine may help maintain immediate performance but may also reduce sleep efficiency, shorten sleep duration, impair subjective sleep quality, and increase next-day sleepiness (Chang et al., 2025; Gardiner et al., 2023; Karakulak et al., 2025; Kocak et al., 2025; Silva et al., 2025). This issue is particularly important during consecutive competitions, tournament-style events, or periods involving high-intensity training on the following day, because the immediate performance benefits may be partly offset by impaired subsequent sleep and recovery.

Therefore, decisions regarding caffeine use should be individualized according to the timing of training or competition, the interval between ingestion and bedtime, habitual caffeine intake, individual sensitivity, and the subsequent training schedule (Guest et al., 2021; Silva et al., 2025). Athletes may test different strategies in non-critical training sessions while simultaneously monitoring exercise performance, perceived fatigue, sleep quality, and recovery status on the following day, rather than using caffeine for the first time during an important competition. For individuals who are sensitive to sleep disruption or who compete in the evening, potential strategies include reducing the dose, increasing the interval between ingestion and bedtime, or avoiding caffeine altogether, while carefully balancing short-term performance demands against the quality of subsequent recovery.

### 4.7 Strengths, limitations, and future directions

A major strength of this study is the evaluation of perceptual, functional, physiological, and sleep recovery within a unified analytical framework. Three-level random-effects models, CR2 cluster-robust inference, prediction intervals, and sensitivity analyses were used to address effect-size dependence and statistical uncertainty. Risk of bias was assessed at the study-by-recovery-domain level using RoB 2, and certainty of evidence was evaluated using GRADE.

Several limitations should nevertheless be acknowledged. First, the number of independent studies and unique participants within individual recovery domains was limited, particularly for sleep recovery, which was represented by only four studies involving 40 unique participants. Moreover, participants were predominantly young men and trained individuals, limiting generalizability to women, adolescents, older adults, and broader physically active populations.

Second, studies varied considerably in caffeine dose, caffeine ingestion timing, exercise model, recovery-assessment time points, and outcome measures. Although moderator analyses were adjusted for recovery domain, many domain-by-moderator combinations contained only one or very few independent studies, precluding reliable interaction analyses. Accordingly, the moderator findings should be considered exploratory and inconclusive rather than evidence that recovery time, dose, ingestion timing or strategy, or exercise model does not modify caffeine effects.

Third, most crossover trials did not report within-participant correlations, requiring assumed values when estimating sampling variances. Although effect directions were generally stable across alternative assumptions and sensitivity analyses, statistical significance for functional and sleep outcomes was sensitive to some analytical assumptions and individual studies. Risk of bias also varied across recovery domains, and several domain-specific assessments were judged to be at high risk, particularly for subjective outcomes that may be susceptible to measurement and expectancy effects.

An additional conceptual limitation concerns the distinction between improved functional performance during recovery and accelerated recovery itself. When caffeine was consumed before exercise or shortly before recovery testing, improvements in strength, jumping, sprinting, or other functional outcomes may partly reflect acute or residual ergogenic, analgesic, or perceptual effects rather than faster restoration of tissue integrity or physiological homeostasis (Matsumura et al., 2026). Functional performance during recovery should therefore not automatically be interpreted as evidence of accelerated recovery. Assessment of publication bias and small-study effects was also limited by the small number of independent studies within each recovery domain. Domain-specific funnel plots and Egger regressions therefore had limited power, and the apparent small-study effect for sleep recovery should be interpreted cautiously because it was based on only four studies.

Future research should prioritize larger, preregistered, and methodologically rigorous randomized controlled trials with greater representation of women and individuals across different ages, training levels, and habitual caffeine-consumption patterns. Studies should clearly distinguish caffeine ingestion before exercise, after exercise, and before recovery testing; standardize reporting of dose and timing; and, where possible, assess perceptual, functional, physiological, and sleep outcomes concurrently. Crossover trials should also report paired data and within-participant correlations.

Particular attention should be given to distinguishing temporary maintenance or enhancement of performance from genuine acceleration of recovery. Longer follow-up is needed to determine effects on next-day performance, consecutive training sessions, sleep, and longer-term adaptation. More balanced coverage across caffeine doses, ingestion strategies and timings, exercise models, and recovery time points will also be necessary to support reliable domain-specific moderator analyses and to clarify how habitual caffeine intake, sex, individual metabolism, and training or competition timing influence the trade-off between short-term performance benefits and subsequent sleep disruption (Pickering and Grgic, 2019; Silva et al., 2025).

## 5 Conclusion

The present study indicates that the effects of caffeine on post-exercise recovery are domain-specific rather than consistent across all dimensions of recovery. Caffeine may produce a small improvement in functional performance during the recovery period, but it does not clearly improve perceptual or physiological recovery and may impair subsequent sleep. The available evidence remains insufficient to determine the optimal dose, caffeine ingestion timing, recovery time window, or exercise model, and uncertainty remains because of the limited number of independent studies and the sensitivity of some findings to analytical assumptions or individual studies. Therefore, caffeine should not be regarded as a supplement that comprehensively enhances post-exercise recovery. Its use should instead be considered in relation to short-term performance demands, individual sensitivity, and subsequent sleep and training schedules. Particular caution is warranted for training and competition in the late afternoon or evening, when potential functional benefits should be carefully weighed against the possibility of subsequent sleep impairment.

## Data Availability

The original contributions presented in the study are included in the article/**Supplementary Material**. Further inquiries can be directed to the corresponding author.
