# Supplementary material for: Effects of caffeine supplementation on post-exercise recovery: a systematic review and three-level meta-analysis of perceptual, functional performance, physiological, and sleep outcomes
Source: Front Physiol. 2026 Sep 17;17:1960237. doi: 10.3389/fphys.2026.1960237 (PMC13626989; doi:10.3389/fphys.2026.1960237)
Supplement: Supplementary file 2 [file Table2.docx]

ELECTRONIC SUPPLEMENTARY MATERIAL CONTENTS

| Number | Material | Page |
| --- | --- | --- |
| 1 | Electronic Supplementary Material Appendix S1 (PRISMA 2020 checklist) | 1-4 |
| 2 | Electronic Supplementary Material Appendix S2 ( Search strategy ) | 5-6 |
| 3 | Electronic Supplementary Material Appendix S3 ( Inclusion and exclusion criteria ) | 7-8 |
| 4 | Electronic Supplementary Material Appendix S4 ( Results of sensitivity analyses ) | 9-11 |
| 5 | Electronic Supplementary Material Appendix S5  (Overall and domain-specific Egger regression results ) | 12-14 |
| 6 | Electronic Supplementary Material Appendix S6 ( GRADE evidence profile ) | 15-17 |
| 7 | Electronic Supplementary Material Appendix S7 ( Characteristics of the included studies ) | 18-22 |
| 8 | Electronic Supplementary Material Appendix S8 ( Risk of Bias in Included Studies ) | 23-27 |
| 9 | Electronic Supplementary Material Appendix S9 ( Heterogeneity estimates) | 27 |

Electronic Supplementary Material Appendix S1 (PRISMA 2020 checklist)

Table S1. PRISMA checklist

| **Section and Topic** | **Item #** | **Checklist item** | **Location where item is reported** |
| --- | --- | --- | --- |
| Effects of Caffeine Supplementation on Post-Exercise Recovery: A Systematic Review and Three-Level Meta-Analysis of Perceptual, Functional, Physiological, and Sleep Outcomes | | |  |
| Title | 1 | Identify the report as a systematic review. | Title page |
| **ABSTRACT** | | |  |
| Abstract | 2 | See the PRISMA 2020 for Abstracts checklist. | Abstract |
| **INTRODUCTION** | | |  |
| Rationale | 3 | Describe the rationale for the review in the context of existing knowledge. | pp. 1–2 |
| Objectives | 4 | Provide an explicit statement of the objective(s) or question(s) the review addresses. | p. 2 |
| **METHODS** | | |  |
| Eligibility criteria | 5 | Specify the inclusion and exclusion criteria for the review and how studies were grouped for the syntheses. | pp. 3–5; Suppl. Table S3 |
| Information sources | 6 | Specify all databases, registers, websites, organisations, reference lists and other sources searched or consulted to identify studies. Specify the date when each source was last searched or consulted. | p. 3 |
| Search strategy | 7 | Present the full search strategies for all databases, registers and websites, including any filters and limits used. | p. 3; Suppl. Table S2 |
| Selection process | 8 | Specify the methods used to decide whether a study met the inclusion criteria of the review, including how many reviewers screened each record and each report retrieved, whether they worked independently, and if applicable, details of automation tools used in the process. | p. 3 |
| Data collection process | 9 | Specify the methods used to collect data from reports, including how many reviewers collected data from each report, whether they worked independently, any processes for obtaining or confirming data from study investigators, and if applicable, details of automation tools used in the process. | p. 4 |
| Data items | 10a | List and define all outcomes for which data were sought. Specify whether all results that were compatible with each outcome domain in each study were sought (e.g. for all measures, time points, analyses), and if not, the methods used to decide which results to collect. | pp. 4–5 |
|  | 10b | List and define all other variables for which data were sought (e.g. participant and intervention characteristics, funding sources). Describe any assumptions made about any missing or unclear information. | pp. 4, 6–7 |
| Study risk of bias assessment | 11 | Specify the methods used to assess risk of bias in the included studies, including details of the tool(s) used, how many reviewers assessed each study and whether they worked independently, and if applicable, details of automation tools used in the process. | pp. 3–4 |
| Effect measures | 12 | Specify for each outcome the effect measure(s) (e.g. risk ratio, mean difference) used in the synthesis or presentation of results. | pp. 5–6 |
| Synthesis methods | 13a | Describe the processes used to decide which studies were eligible for each synthesis (e.g. tabulating the study intervention characteristics and comparing against the planned groups for each synthesis (item #5)). | pp. 4–5, 8 |
|  | 13b | Describe any methods required to prepare the data for presentation or synthesis, such as handling of missing summary statistics, or data conversions. | pp. 6–7 |
|  | 13c | Describe any methods used to tabulate or visually display results of individual studies and syntheses. | Not explicit; Figs. 1–9, pp. 11–23 |
|  | 13d | Describe any methods used to synthesize results and provide a rationale for the choice(s). If meta-analysis was performed, describe the model(s), method(s) to identify the presence and extent of statistical heterogeneity, and software package(s) used. | pp. 7–8 |
|  | 13e | Describe any methods used to explore possible causes of heterogeneity among study results (e.g. subgroup analysis, meta-regression). | p. 8 |
|  | 13f | Describe any sensitivity analyses conducted to assess robustness of the synthesized results. | pp. 8–9 |
| Reporting bias assessment | 14 | Describe any methods used to assess risk of bias due to missing results in a synthesis (arising from reporting biases). | p. 9 |
| Certainty assessment | 15 | Describe any methods used to assess certainty (or confidence) in the body of evidence for an outcome. | pp. 9–10 |
| **RESULTS** | | |  |
| Study selection | 16a | Describe the results of the search and selection process, from the number of records identified in the search to the number of studies included in the review, ideally using a flow diagram. | pp. 10–11; Fig. 1 |
|  | 16b | Cite studies that might appear to meet the inclusion criteria, but which were excluded, and explain why they were excluded. | Not reported |
| Study characteristics | 17 | Cite each included study and present its characteristics. | p. 12; Suppl. Table S7 |
| Risk of bias in studies | 18 | Present assessments of risk of bias for each included study. | p. 12; Suppl. Figs. S2A–S2B; Tables S8A–S8B |
| Results of individual studies | 19 | For all outcomes, present, for each study: (a) summary statistics for each group (where appropriate) and (b) an effect estimate and its precision (e.g. confidence/credible interval), ideally using structured tables or plots. | Figs. 2–5, pp. 13–17; Suppl. Data |
| Results of syntheses | 20a | For each synthesis, briefly summarise the characteristics and risk of bias among contributing studies. | pp. 12–17; Suppl. Tables S7–S9 |
|  | 20b | Present results of all statistical syntheses conducted. If meta-analysis was done, present for each the summary estimate and its precision (e.g. confidence/credible interval) and measures of statistical heterogeneity. If comparing groups, describe the direction of the effect. | pp. 13–21; Figs. 2–8; Suppl. Table S9 |
|  | 20c | Present results of all investigations of possible causes of heterogeneity among study results. | pp. 17–21; Figs. 6–8 |
|  | 20d | Present results of all sensitivity analyses conducted to assess the robustness of the synthesized results. | pp. 21–22; Suppl. Table S4 |
| Reporting biases | 21 | Present assessments of risk of bias due to missing results (arising from reporting biases) for each synthesis assessed. | pp. 22–23; Fig. 9; Suppl. Table S5; Figs. S1A–S1B |
| Certainty of evidence | 22 | Present assessments of certainty (or confidence) in the body of evidence for each outcome assessed. | pp. 23–24; Suppl. Table S6 |
| **DISCUSSION** | | |  |
| Discussion | 23a | Provide a general interpretation of the results in the context of other evidence. | pp. 24–29 |
|  | 23b | Discuss any limitations of the evidence included in the review. | pp. 30–31 |
|  | 23c | Discuss any limitations of the review processes used. | pp. 30–31 |
|  | 23d | Discuss implications of the results for practice, policy, and future research. | pp. 29–31 |
| **OTHER INFORMATION** | | |  |
| Registration and protocol | 24a | Provide registration information for the review, including register name and registration number, or state that the review was not registered. | Abstract; p. 3 |
|  | 24b | Indicate where the review protocol can be accessed, or state that a protocol was not prepared. | p. 3 |
|  | 24c | Describe and explain any amendments to information provided at registration or in the protocol. | No amendments reported |
| Support | 25 | Describe sources of financial or non-financial support for the review, and the role of the funders or sponsors in the review. | p. 33 |
| Competing interests | 26 | Declare any competing interests of review authors. | p. 33 |
| Availability of data, code and other materials | 27 | Report which of the following are publicly available and where they can be found: template data collection forms; data extracted from included studies; data used for all analyses; analytic code; any other materials used in the review. | pp. 32–33 |

Electronic Supplementary Material Appendix S2 ( Search strategy )

Table S2. Search strategy

Panel A. Electronic database search strategies

| Database | retrieval strategy |
| --- | --- |
| **WOS** | #1 TS=( caffeine OR coffee OR "caffeine supplementation" OR "caffeine ingestion" OR "caffeinated beverage" OR "energy drink" )  #2 TS=( recovery OR "post-exercise recovery" OR "exercise recovery" OR "muscle recovery" OR fatigue OR pain OR soreness OR "delayed onset muscle soreness" OR DOMS OR "exercise-induced muscle damage" OR EIMD OR "muscle damage" OR "muscle function" OR "muscle strength" OR power OR "creatine kinase" OR CK OR "heart rate variability" OR HRV OR glycogen OR sleep OR "sleep quality" OR "sleep efficiency" )  #3 TS=( exercise OR training OR sport* OR athlete* OR "resistance exercise" OR "resistance training" OR "endurance exercise" OR cycling OR running OR sprint* OR "high-intensity exercise" )  #4 TS=( randomized OR randomised OR placebo OR trial OR crossover OR "cross-over" OR "double-blind" )  #5 #1 AND #2 AND #3 AND #4 |
| **Embase** | #1 'caffeine'/exp OR 'coffee'/exp OR caffeine:ti,ab,kw OR coffee:ti,ab,kw OR 'caffeine supplementation':ti,ab,kw OR 'caffeine ingestion':ti,ab,kw OR 'caffeinated beverage':ti,ab,kw OR 'energy drink':ti,ab,kw  #2 'muscle fatigue'/exp OR 'muscle soreness'/exp OR 'muscle injury'/exp OR 'creatine kinase'/exp OR 'heart rate variability'/exp OR 'glycogen'/exp OR 'sleep'/exp OR recovery:ti,ab,kw OR 'post-exercise recovery':ti,ab,kw OR 'exercise recovery':ti,ab,kw OR 'muscle recovery':ti,ab,kw OR fatigue:ti,ab,kw OR soreness:ti,ab,kw OR 'delayed onset muscle soreness':ti,ab,kw OR doms:ti,ab,kw OR 'exercise-induced muscle damage':ti,ab,kw OR eimd:ti,ab,kw OR 'muscle damage':ti,ab,kw OR 'muscle function':ti,ab,kw OR 'muscle strength':ti,ab,kw OR 'creatine kinase':ti,ab,kw OR 'heart rate variability':ti,ab,kw OR hrv:ti,ab,kw OR glycogen:ti,ab,kw OR sleep:ti,ab,kw OR 'sleep quality':ti,ab,kw OR 'sleep efficiency':ti,ab,kw  #3 'exercise'/exp OR 'exercise training'/exp OR 'sport'/exp OR exercise:ti,ab,kw OR training:ti,ab,kw OR sport*:ti,ab,kw  OR athlete*:ti,ab,kw OR 'resistance exercise':ti,ab,kw OR 'resistance training':ti,ab,kw OR 'endurance exercise':ti,ab,kw OR cycling:ti,ab,kw OR running:ti,ab,kw OR sprint*:ti,ab,kw OR 'high-intensity exercise':ti,ab,kw  #4 'randomized controlled trial'/exp OR 'crossover procedure'/exp OR 'double blind procedure'/exp OR randomized:ti,ab,kw OR randomised:ti,ab,kw OR placebo:ti,ab,kw OR trial:ti,ab,kw OR crossover:ti,ab,kw OR 'cross-over':ti,ab,kw OR 'double-blind':ti,ab,kw  #5 #1 AND #2 AND #3 AND #4 |
| **Pubmed** | #1 caffeine[Title/Abstract] OR coffee[Title/Abstract] OR "caffeine supplementation"[Title/Abstract] OR "caffeine ingestion"[Title/Abstract] OR "caffeinated beverage"[Title/Abstract] OR "energy drink"[Title/Abstract]  #2 recovery[Title/Abstract] OR "post-exercise recovery"[Title/Abstract] OR "exercise recovery"[Title/Abstract] OR "muscle recovery"[Title/Abstract] OR fatigue[Title/Abstract] OR pain[Title/Abstract] OR soreness[Title/Abstract] OR "delayed onset muscle soreness"[Title/Abstract] OR DOMS[Title/Abstract] OR "exercise-induced muscle damage"[Title/Abstract] OR EIMD[Title/Abstract] OR "muscle damage"[Title/Abstract] OR "muscle function"[Title/Abstract] OR "muscle strength"[Title/Abstract] OR power[Title/Abstract] OR "creatine kinase"[Title/Abstract] OR CK[Title/Abstract] OR "heart rate variability"[Title/Abstract] OR HRV[Title/Abstract] OR glycogen[Title/Abstract] OR sleep[Title/Abstract] OR "sleep quality"[Title/Abstract] OR "sleep efficiency"[Title/Abstract]  #3 exercise[Title/Abstract] OR training[Title/Abstract] OR sport[Title/Abstract] OR "resistance exercise"[Title/Abstract] OR "resistance training"[Title/Abstract] OR "endurance exercise"[Title/Abstract] OR cycling[Title/Abstract] OR running[Title/Abstract] OR sprint[Title/Abstract] OR "high-intensity exercise"[Title/Abstract]  #4 randomized[Title/Abstract] OR randomised[Title/Abstract] OR placebo[Title/Abstract] OR trial[Title/Abstract] OR crossover[Title/Abstract] OR "cross-over"[Title/Abstract] OR "double-blind"[Title/Abstract]  #5 #1 AND #2 AND #3 AND #4 |
| **Cochrane Library** | #1 MeSH descriptor: [Caffeine] explode all trees OR caffeine:ti,ab,kw OR coffee:ti,ab,kw OR "energy drink":ti,ab,kw  #2 recovery:ti,ab,kw OR "muscle recovery":ti,ab,kw OR "exercise-induced muscle damage":ti,ab,kw OR DOMS:ti,ab,kw OR soreness:ti,ab,kw OR "creatine kinase":ti,ab,kw OR "heart rate variability":ti,ab,kw OR glycogen:ti,ab,kw OR sleep:ti,ab,kw  #3 MeSH descriptor: [Exercise] explode all trees OR exercise:ti,ab,kw OR training:ti,ab,kw OR sport*:ti,ab,kw OR athlete*:ti,ab,kw OR "resistance exercise":ti,ab,kw OR "endurance exercise":ti,ab,kw  #4 randomized:ti,ab,kw OR randomised:ti,ab,kw OR placebo:ti,ab,kw OR trial:ti,ab,kw OR crossover:ti,ab,kw OR "cross-over":ti,ab,kw OR "double-blind":ti,ab,kw  #5 #1 AND #2 AND #3 AND #4 |
| **SPORTDiscus** | #1 caffeine OR coffee OR "caffeine supplementation" OR "caffeine ingestion" OR "caffeinated beverage" OR "energy drink"  #2 recovery OR "post-exercise recovery" OR "exercise recovery" OR "muscle recovery" OR fatigue OR pain OR soreness OR "exercise-induced muscle damage" OR EIMD OR DOMS OR "delayed onset muscle soreness" OR "creatine kinase" OR CK OR "heart rate variability" OR HRV OR glycogen OR sleep  #3 exercise OR training OR sport* OR athlete* OR "resistance exercise" OR "resistance training" OR "endurance exercise" OR cycling OR running OR sprint* OR "high-intensity exercise"  #4 randomized OR randomised OR placebo OR trial OR crossover OR "cross-over" OR "double-blind"  #5 #1 AND #2 AND #3 AND #4 |

Panel B. Supplementary search methods

| **Source** | **Search details** |
| --- | --- |
| **Google Scholar** | Search conducted on June 30, 2026. The following search string was used: “caffeine” AND exercise AND (recovery OR “post-exercise recovery” OR “muscle soreness” OR DOMS OR “muscle damage” OR “creatine kinase” OR sleep) AND (placebo OR randomized OR crossover). Results were sorted by relevance and screened sequentially. |
| **ResearchGate** | Search conducted on June 30, 2026 using the following keyword combinations: “caffeine exercise recovery”, “caffeine muscle soreness recovery”, “caffeine muscle damage recovery”, and “caffeine exercise sleep”. Results were screened in order of relevance. |
| **Reference-list screening** | The reference lists of all included studies and relevant systematic reviews and meta-analyses were manually screened for potentially eligible primary studies. |

Electronic Supplementary Material Appendix S3 (Inclusion and exclusion criteria )

Table S3. Inclusion and exclusion criteria.

Inclusion and Exclusion Criteria

Inclusion criteria

(1) Participants

Participants were healthy human individuals who engaged in an exercise or training task, including competitive athletes, trained individuals, physically active individuals, and healthy members of the general population. No restrictions were imposed on sex or training status. Studies were required to report basic participant characteristics, such as sample size, age, sex, and/or training background.

(2) Intervention

The intervention consisted of caffeine supplementation or the consumption of a caffeine-containing product, including capsules, beverages, coffee, or any other form for which the caffeine dose could be clearly determined. Caffeine could be consumed before, during, or after exercise, provided that the study evaluated its effects on post-exercise recovery or subsequent sleep. For multi-ingredient products containing other active substances, studies were included only when the independent effect of caffeine could be distinguished.

(3) Comparator

Eligible comparators included placebo, caffeine-free conditions, decaffeinated products, or control products matched to the caffeine condition in appearance, taste, and other ingredients. For studies with multiple intervention arms, only data allowing a direct comparison between an eligible caffeine condition and an eligible control condition were extracted.

(4) Outcomes

Studies were required to report at least one recovery-related outcome measured after exercise or during the subsequent sleep period. Recovery outcomes were classified into the following four domains:

Perceptual recovery: delayed-onset muscle soreness, muscle pain, perceived fatigue, perceived recovery status, tenderness, pressure pain threshold, or other related perceptual outcomes;

Functional performance during recovery: maximal voluntary isometric contraction, maximal voluntary contraction, isokinetic peak torque, jump performance, sprint performance, endurance performance, or other indices of physical function measured during the post-exercise recovery period;

Physiological recovery: creatine kinase, lactate dehydrogenase, myoglobin, aspartate aminotransferase, alanine aminotransferase, oxidative stress markers, or other post-exercise physiological indicators;

Sleep recovery: total sleep time, sleep efficiency, sleep onset latency, wake after sleep onset, number of awakenings, sleep stages, subjective sleep quality, or other related sleep outcomes.

Studies meeting all other eligibility criteria were retained in the qualitative synthesis even when the reported numerical data were insufficient for effect-size calculation. For inclusion in the meta-analysis, studies were required to provide sufficient data for effect-size calculation, including sample sizes, means and standard deviations, change scores and their measures of dispersion, standard errors, confidence intervals, or data that could be derived from figures or other reported statistical information.

(5) Study design

Each study was required to include at least one caffeine intervention condition and one placebo, caffeine-free, or other appropriate control condition, and to report relevant outcomes during the post-exercise recovery period or subsequent sleep period.

Exclusion criteria

(1) Ineligible participants

Animal studies, in vitro studies, and studies primarily focused on clinical treatment or disease rehabilitation were excluded. Studies involving participants with medical conditions that could substantially affect caffeine metabolism, exercise responses, or recovery processes were also excluded when data for healthy participants could not be extracted separately.

(2) Independent effects of caffeine could not be determined

Studies that did not clearly report caffeine intake were excluded. Studies in which caffeine was combined with carbohydrates, protein, creatine, or other active ingredients were also excluded when the control condition was not matched for the same co-interventions, thereby preventing the independent effect of caffeine from being isolated.

(3) Absence of an eligible control condition

Studies without a placebo, caffeine-free, or other appropriate control condition were excluded. Studies that compared only different caffeine doses, caffeine ingestion timings or strategies, or caffeine-containing products without an eligible control condition were also excluded.

(4) Post-exercise recovery was not assessed

Studies that evaluated only acute exercise performance, physiological responses, or perceptual responses during exercise, without reporting post-exercise recovery outcomes or subsequent sleep outcomes, were excluded. For example, studies reporting only repetitions completed, training volume, heart rate, blood lactate concentration, rating of perceived exertion, or performance during the initial exercise test were not eligible.

(5) Ineligible data or study type

Non-randomized trials, observational studies, cross-sectional studies, case reports, reviews, meta-analyses, conference abstracts, theses or dissertations, book chapters, commentaries, methodological studies, and expert opinions were excluded. Studies reporting eligible recovery outcomes but lacking sufficient numerical data for effect-size calculation were excluded from the quantitative meta-analysis but could be retained in the qualitative synthesis if all other eligibility criteria were met. For duplicate publications or studies with clearly overlapping samples, the version with the most complete information, the largest sample size, or the most comprehensive outcome reporting was preferentially included.

Electronic Supplementary Material Appendix S4 (Results of sensitivity analyses )

Table S4. Sensitivity analyses of the primary recovery-domain meta-analyses

Panel A. General sensitivity analyses across recovery domains

| Outcome | Main analysis, g [CR2 95% CI] | Main CR2 p | r = 0.30,  [CR2 95% CI] | r = 0.30  CR2 p | r = 0.50,  g [CR2 95% CI] | r = 0.50  CR2 p | r = 0.70,  g [CR2 95% CI] | r = 0.70  CR2 p | Leave-one-study-out g range | Leave-one-study-out CR2 p range | Domain-specific high-risk studies excluded | After exclusion, g [CR2 95% CI] | After exclusion CR2 p | Interpretation |
| --- | --- | --- | --- | --- | --- | --- | --- | --- | --- | --- | --- | --- | --- | --- |
| Perceptual recovery | −0.161  [−0.528, 0.205] | 0.319 | −0.166  [−0.539, 0.207] | 0.314 | −0.161  [−0.528, 0.205] | 0.319 | −0.157  [−0.519, 0.205] | 0.324 | −0.236 to −0.069 | 0.192  to 0.621 | Filip-Stachnik et al. (2023);  Maridakis et al. (2007);  Hurley et al. (2013) | −0.159  [−1.034, 0.716] | 0.603 | Across different r assumptions, leave-one-study-out analyses, and analyses excluding studies judged to be at high risk of bias within the perceptual recovery domain, the direction of effect and the overall non-significant conclusion remained largely stable. |
| Functional performance during recovery | −0.208  [−0.393, −0.023] | 0.035 | −0.199  [−0.412, 0.015] | 0.060 | −0.208  [−0.393, −0.023] | 0.035 | −0.215  [−0.392, −0.037] | 0.025 | -0.264  to -0.157 | 0.017  to 0.080 | Caldwell  et al. (2017)；Maridakis  et al. (2007) | −0.203 [−0.405, −0.002] | 0.049 | The direction of effect consistently favoured caffeine; however, statistical significance varied under the r = 0.30 assumption and after the omission of individual studies. The finding therefore showed greater stability in effect direction than in statistical significance. |
| Physiological recovery | −0.104  [−0.346, 0.138] | 0.293 | −0.104  [−0.353, 0.146] | 0.307 | −0.104  [−0.346, 0.138] | 0.293 | −0.105  [−0.345, 0.135] | 0.284 | -0.151  to -0.028 | 0.219  to 0.416 | Filip-Stachnik et al. (2023) | −0.117  [−0.492, 0.259] | 0.390 | Across the sensitivity analyses, the direction of effect and the overall non-significant conclusion remained generally stable. |
| Sleep recovery | 0.751  [0.244, 1.258] | 0.018 | 0.752  [0.245, 1.259] | 0.018 | 0.751  [0.244, 1.258] | 0.018 | 0.750  [0.243, 1.257] | 0.018 | 0.683  to 0.890 | 0.007  to 0.085 | None | Not applicable | Not applicable | The adverse direction remained stable across different r assumptions and leave-one-study-out analyses, although statistical significance varied after the omission of individual studies. No study was rated as being at high risk of bias within the sleep recovery domain. |

*Note: Effect sizes are presented as Hedges’g with CR2 robust 95% confidence intervals (CIs). Negative values favour caffeine, whereas positive values favour placebo. The primary analysis assumed a within-participant correlation of r = 0.50 for crossover trials, with sensitivity analyses conducted using r = 0.30 and 0.70. Pooled point estimates were obtained from three-level random-effects models using restricted maximum likelihood estimation, whereas 95% CIs and p values were obtained using CR2 cluster-robust variance estimation with study as the clustering unit and Satterthwaite small-sample degrees-of-freedom correction. The ranges reported for the leave-one-study-out analyses represent the minimum and maximum pooled effect estimates and CR2 p values obtained after sequentially excluding each study. Studies judged to be at high risk of bias within the corresponding recovery domain were identified based on the study-by-recovery-domain RoB 2 assessments. CI, confidence interval; CR2, bias-reduced cluster-robust variance estimation.*

Panel B. Additional sensitivity analyses for functional performance addressing potential acute or residual ergogenic effects

| Analysis | Effect sizes, k | Studies, n | Hedges’*g* [CR2 95% CI] | CR2 p | Interpretation |
| --- | --- | --- | --- | --- | --- |
| Primary functional-domain analysis | 30 | 9 | −0.208 [−0.393, −0.023] | 0.035 | Small effect favouring caffeine |
| Excluding functional assessments ≤1 h | 28 | 8 | −0.153 [−0.353, 0.048] | 0.089 | Direction remained favourable to caffeine, but the effect was attenuated and no longer statistically significant |
| Excluding outcomes with caffeine ingestion before recovery testing | 3 | 3 | −0.548 [−2.048, 0.952] | 0.233 | Estimate remained favourable to caffeine but was highly imprecise because only three studies remained |
| Excluding assessments ≤1 h or caffeine ingestion before recovery testing | 2 | 2 | −0.448 [−6.547, 5.650] | 0.522 | Direction remained favourable to caffeine, but the evidence was too sparse for reliable inference |

*Note: Effect sizes are presented as Hedges’g with CR2 robust 95% confidence intervals (CIs). Negative values favour caffeine, whereas positive values favour placebo. Assessments performed within 1 h after exercise and outcomes for which caffeine was ingested specifically before the recovery test were excluded separately and jointly to evaluate the potential influence of acute or residual ergogenic effects on functional performance during recovery. Pooled point estimates were obtained from three-level random-effects models using restricted maximum likelihood estimation, whereas 95% CIs and p values were obtained using CR2 cluster-robust variance estimation with study as the clustering unit and Satterthwaite small-sample degrees-of-freedom correction. k, number of effect sizes; n, number of independent studies; CI, confidence interval; CR2, bias-reduced cluster-robust variance estimation.*

| Domain | Primary analysis g [CR2 95% CI] | One-study-one-domain g [CR2 95% CI] | CR2 p | Δg |
| --- | --- | --- | --- | --- |
| Perceptual | −0.161 [−0.528, 0.205] | −0.198 [−0.639, 0.242] | 0.312 | −0.037 |
| Functional | −0.208 [−0.393, −0.023] | −0.248 [−0.466, −0.029] | 0.032 | −0.040 |
| Physiological | −0.104 [−0.346, 0.138] | −0.143 [−0.431, 0.146] | 0.226 | −0.039 |
| Sleep | 0.751 [0.244, 1.258] | 0.782 [0.207, 1.357] | 0.024 | +0.031 |

Panel C. One-study-one-domain sensitivity analysis

*Note: The one-study-one-domain sensitivity analysis reduced each study to a single composite effect size within each recovery domain, thereby preventing studies contributing multiple outcomes or time points from receiving disproportionate influence because of denser reporting. Within each study-by-domain combination, effect sizes were combined using an equal-weight approach under a conservative working correlation of ρ = 1.00. The resulting composite effects were then pooled using random-effects meta-analysis. Negative values favour caffeine, whereas positive values favour placebo. Δg represents the difference between the one-study-one-domain estimate and the corresponding primary three-level meta-analytic estimate. CI, confidence interval; CR2, bias-reduced cluster-robust variance estimation.*

Electronic Supplementary Material Appendix S5

(Overall and domain-specific Egger regression results )

Table S5. Results of the overall and domain-specific three-level Egger regression analyses.

| Analysis | k | No. of studies | Egger β | CR2 95% CI | CR2 *p* value | Interpretation |
| --- | --- | --- | --- | --- | --- | --- |
| Overall (domain-adjusted) | 120 | 16 | −0.670 | −5.748 to 4.408 | 0.753 | No clear evidence of small-study effects |
| Perceptual recovery | 26 | 7 | −7.789 | −48.190 to 32.612 | 0.297 | No clear evidence of small-study effects |
| Functional performance during recovery | 30 | 9 | −2.696 | −8.692 to 3.301 | 0.155 | No clear evidence of small-study effects |
| Physiological recovery | 36 | 5 | −9.666 | −39.054 to 19.723 | 0.182 | No clear evidence of small-study effects |
| Sleep recovery | 28 | 4 | 8.488 | 2.007 to 14.970 | 0.029 | Possible small-study effects |

*Note: The overall analysis used a domain-adjusted three-level Egger regression, whereas the domain-specific analyses were conducted using exploratory three-level Egger regressions. Statistical inference was based on CR2 cluster-robust variance estimation with study as the clustering unit and Satterthwaite small-sample degrees-of-freedom correction; accordingly, the reported 95% confidence intervals and p values are CR2 robust estimates. Because each recovery domain included fewer than 10 independent studies, the domain-specific analyses were considered exploratory and should be interpreted cautiously. A non-significant Egger test should not be interpreted as evidence that publication bias is absent, and the statistically significant result for sleep recovery should not be regarded as definitive evidence of publication bias. k, number of effect sizes; CI, confidence interval; CR2, bias-reduced cluster-robust variance estimation.*

Figure S1A. Contour-enhanced funnel plots for the four recovery domains

*Note: Each circle represents an individual effect size. The shaded regions indicate different levels of statistical significance, and the vertical dashed line represents the line of no effect. Domain-specific Egger regressions were performed using three-level models with CR2 cluster-robust inference and Satterthwaite small-sample degrees-of-freedom correction. Because each recovery domain included fewer than 10 independent studies, these analyses were considered exploratory and should be interpreted cautiously. Non-significant Egger tests should not be interpreted as evidence that publication bias is absent, and the statistically significant result for sleep recovery should not be regarded as definitive evidence of publication bias.*

Figure S1B. Power-enhanced funnel plots for the four recovery domains

*Note: The background colours represent the theoretical statistical power of an individual effect size at different levels of standard error, assuming that the true effect equals the pooled estimate from the corresponding domain-specific three-level model. Red indicates lower power, whereas green indicates higher power. The thick black lines denote the 95% funnel boundaries centred on the null effect, and the black shaded bands indicate the regions between the 90% and 95% boundaries. The thin funnel boundaries and solid vertical lines are centred on the pooled effect estimate for each domain. Circles represent individual effect sizes. Domain-specific Egger regressions were conducted using three-level models with CR2 cluster-robust inference. Because each recovery domain included fewer than 10 independent studies, these analyses were considered exploratory and should be interpreted cautiously. The power-enhanced funnel plots were used to describe theoretical statistical power and the distribution of low-powered effect sizes and were not used as an independent criterion for determining publication bias.*

Electronic Supplementary Material Appendix S6 ( GRADE evidence profile )

Table S6. GRADE evidence profile for the effects of caffeine supplementation on post-exercise recovery outcomes.

| Outcome domain | No. of studies | No. of effect sizes | No. of unique participants | Hedges’*g*  （CR2 95% CI） | CR2  *P* value | 95% prediction interval (model-based) | Risk of bias | Inconsistency | Indirectness | Imprecision | Publication  bias | Certainty  of evidence | Conclusion |
| --- | --- | --- | --- | --- | --- | --- | --- | --- | --- | --- | --- | --- | --- |
| Perceptual recovery | 7 | 26 | 86 | −0.161  (−0.528 to 0.205) | 0.319 | −0.980 to 0.658 | Serious | Serious | Not serious | Serious | No additional downgrade | Very low  ⊕OOO | The evidence is very uncertain regarding the effect of caffeine on perceptual recovery. [a, e, f, j] |
| Functional performance during recovery | 9 | 30 | 125 | −0.208  (−0.393 to −0.023) | 0.035 | −0.444 to 0.028 | Not serious | Not serious | Not serious | Serious | No additional downgrade | Moderate  ⊕⊕⊕O | Moderate-certainty evidence suggests that caffeine may produce a small beneficial effect on functional performance during recovery, although the magnitude of the effect remains uncertain. [b, g, j] |
| Physiological recovery | 5 | 36 | 66 | −0.104  (−0.346 to 0.138) | 0.293 | −0.492 to 0.284 | Not serious | Not serious | Not serious | Serious | No additional downgrade | Moderate  ⊕⊕⊕O | Moderate-certainty evidence suggests that caffeine probably has little or no clear effect on physiological recovery markers. [c, h, j] |
| Sleep recovery | 4 | 28 | 40 | 0.751  (0.244 to 1.258) | 0.018 | −0.002 to 1.503 | Serious | Not serious | Not serious | Serious | No additional downgrade | Low  ⊕⊕OO | Low-certainty evidence suggests that caffeine may impair post-exercise sleep recovery, although the magnitude and consistency of the adverse effect remain uncertain. [d, i, j] |

GRADE downgrading rationale and explanatory footnotes

| GRADE downgrading footnotes | |
| --- | --- |
| a. Risk of bias — perceptual recovery | The certainty of evidence for perceptual recovery was downgraded by one level for risk of bias. Based on the study-by-recovery-domain RoB 2 assessments, three of the seven studies contributing to perceptual recovery were judged to be at high risk of bias. After exclusion of these studies, only four studies and eight effect sizes remained. Although the pooled point estimate was almost unchanged (Hedges’ g = −0.159), the substantially reduced evidence base increased concern regarding the influence of study limitations on this domain. |
| b. Risk of bias — functional performance during recovery | The certainty of evidence for functional performance during recovery was not downgraded for risk of bias. Two of the nine studies were judged to be at high risk of bias for the functional-performance domain. After excluding Caldwell et al. (2017) and Maridakis et al. (2007), the pooled estimate remained small and continued to favour caffeine (Hedges’ g = −0.203; CR2 p = 0.049), with little change in the magnitude or direction of the effect. |
| c. Risk of bias — physiological recovery | The certainty of evidence for physiological recovery was not downgraded for risk of bias. One of the five studies was judged to be at high risk of bias for the physiological recovery domain. After excluding Filip-Stachnik et al. (2023), the pooled point estimate remained similar and the direction of effect was unchanged (Hedges’ g = −0.117; CR2 p = 0.390), indicating that the primary conclusion was not materially altered. |
| d. Risk of bias — sleep recovery | The certainty of evidence for sleep recovery was downgraded by one level for risk of bias. All four studies contributing to this domain were judged as having some concerns in the study-by-recovery-domain RoB 2 assessments, and none was rated as being at low risk of bias. The principal concerns related to outcome measurement, particularly for subjective sleep outcomes, and potential selective reporting. |
| e. Inconsistency — perceptual recovery | The certainty of evidence for perceptual recovery was downgraded by one level for inconsistency. Total heterogeneity was I² = 60.08%, comprising Level 3 between-study heterogeneity of I² = 37.78% and Level 2 within-study heterogeneity of I² = 22.30%. The model-based 95% prediction interval ranged from −0.980 to 0.658 and encompassed beneficial, near-null, and adverse effects, indicating that the true effect may vary meaningfully across study settings. |
| f. Imprecision — perceptual recovery | The certainty of evidence for perceptual recovery was downgraded by one level for imprecision. Seven independent studies involving 86 unique participants contributed to this domain, and the CR2 95% confidence interval was wide (−0.528 to 0.205), encompassing a potentially meaningful beneficial effect, a near-null effect, and a possible adverse effect. The available evidence therefore does not provide a sufficiently precise estimate of the effect of caffeine on perceptual recovery. |
| g. Imprecision — functional performance during recovery | The certainty of evidence for functional performance during recovery was downgraded by one level for imprecision. Nine independent studies involving 125 unique participants contributed to this domain. Although the CR2 95% confidence interval did not cross the null (−0.393 to −0.023), it ranged from an effect close to the null to a more appreciable beneficial effect. In addition, statistical significance varied across sensitivity analyses, indicating uncertainty regarding the precise magnitude and stability of the functional effect. |
| h. Imprecision — physiological recovery | The certainty of evidence for physiological recovery was downgraded by one level for imprecision. Only five independent studies involving 66 unique participants contributed to this domain, and the CR2 95% confidence interval ranged from −0.346 to 0.138. This interval encompassed potentially beneficial effects, effects close to the null, and possible adverse effects, resulting in substantial uncertainty regarding the magnitude and direction of the true effect. |
| i. Imprecision — sleep recovery | The certainty of evidence for sleep recovery was downgraded by one level for imprecision. Only four independent studies involving 40 unique participants contributed to this domain. Although the pooled effect indicated poorer sleep recovery with caffeine, the CR2 95% confidence interval was wide (0.244 to 1.258), and the model-based 95% prediction interval ranged from −0.002 to 1.503, extending to approximately the null. In addition, the statistical significance of the sleep effect showed some sensitivity to alternative analytical assumptions and individual studies. Taken together, these findings indicate substantial uncertainty regarding the magnitude and consistency of the adverse sleep effect. |
| j. Publication bias — all recovery domains | Publication bias could not be reliably assessed because fewer than 10 independent studies contributed to each recovery domain. The non-significant exploratory Egger tests for perceptual, functional, and physiological recovery were not interpreted as evidence that publication bias was absent. For sleep recovery, the exploratory Egger regression suggested possible small-study effects (CR2 p = 0.029); however, only four independent studies contributed to this domain, making the estimate highly unstable. Accordingly, the possibility of publication bias could not be excluded in any domain, but the available evidence was considered insufficient to justify an additional downgrade for publication bias. |

Electronic Supplementary Material Appendix S7 (Characteristics of the included studies)

Table S7. Characteristics of the included studies

| Study | Design / sample | Participants | Exercise /recovery model | Caffeine intervention | Recovery assessment  time points | Outcomes/ domains | Main findings and  meta-analysis notes |
| --- | --- | --- | --- | --- | --- | --- | --- |
| Machado et al.  (2009) | Parallel-group design; n = 15  (CAF = 8, PLA = 7) | Male soccer players;  18 ± 3 years | Soccer-specific intermittent sprint protocol;  two trials consisting of  6 × 10 × 20-m sprints | 5.5 mg/kg; capsules; administered 45 min before exercise; single dose | PRE ,24, 48, and 72 h | CK, LDH, AST, and ALT [Physiological] | CAF did not significantly alter enzymatic markers compared with PLA; data were eligible for the analysis of biochemical outcomes. |
| Filip-Stachnik et al.  (2023) | Crossover design;  n = 10; CAF/PLA | Resistance-trained men;  24 ± 7 years | Whole-body resistance exercise involving seven exercises performed at 70% 1RM to failure | 3 mg/kg; capsules; administered 60 min before exercise; single dose | Before supplementation, after supplementation, immediately post-exercise,  1 h, and 24 h | Training performance [Functional]; RPE, fatigue, and soreness [Perceptual]; CK, LDH, and oxidative stress markers [Physiological] | CAF did not improve training performance, RPE, CK, or LDH, although some oxidative stress markers were favourably affected. |
| AbuMoh’d et al.  (2021) | Crossover design;  n = 15; CAF/PLA | Trained men;  30.7 ± 3.4 years | Resistance exercise followed by an incremental treadmill test to exhaustion | 6 mg/kg; capsules; administered 60 min before testing; single dose | PRE, POST, and 24 h; time to exhaustion assessed separately | CK, LDH, CK-MB, and myoglobin [Physiological];  time to exhaustion [Functional] | CAF did not substantially improve muscle-damage biomarkers but prolonged time to exhaustion. |
| Green et al.  (2018) | Crossover design;  n = 16; CAF/PLA | Physically active adults;  8 men and 8 women;  24.3 ± 4.3 years | Quadriceps eccentric muscle-damage protocol consisting of 10 × 10 eccentric leg extensions | 6 mg/kg; capsules; administered 30 min before functional testing;  single dose | Immediately and 24 h after muscle-damaging exercise | MVIC, RTD, and fatigue index [Functional];  quadriceps soreness [Perceptual] | CAF improved some indices of muscle strength but had little effect on soreness or fatigue index. |
| Machado et al.  (2008) | Crossover design;  n = 15; CAF/PLA | Male soccer players;  18.4 ± 0.8 years | Whole-body resistance exercise involving seven exercises performed for 3 × 10RM | Approximately 4.5 mg/kg; capsules; administered after the PRE assessment;  single dose | PRE, 24 h | CK, LDH, AST, and ALT [Physiological] | CAF did not significantly alter biochemical markers of muscle damage compared with PLA. |
| Al-Nawaiseh et al.  (2022) | Crossover design;  n = 11; CAF/PLA | Recreational runners; 9 men and 2 women; 24.5 ± 6.3 years | Downhill running to induce DOMS, followed by a 5-km time trial 48 h later | 5 mg/kg; capsules; administered 60 min before the 5-km test; single dose | Immediately, 24 h, and 48 h after downhill running; before and after the 5-km test | Soreness VAS, pressure pain threshold, and RPE [Perceptual]; HR [Physiological];  5-km performance [Functional] | CAF did not improve 5-km performance or RPE, and perceived soreness was higher under the CAF condition. |
| Caldwell et al.  (2017) | Parallel-group design; n = 30  (CAF = 12, PLA = 18) | Cyclists; 25 men and 5 women | A 164-km endurance cycling event | 3 mg/kg; tablets; administered immediately after cycling and repeatedly during recovery | Before and after the event and on recovery days 1–4 | General and leg soreness [Perceptual]; lower-extremity function [Functional] | CAF may have reduced some soreness ratings; however, sample sizes varied across outcomes. |
| Bezuglov et al.  (2025) | Parallel-group design; four groups;  n = 54  (CAF = 27, PLA = 27) | Elite youth male soccer players; median age, 16.5 years | Soccer-specific high-intensity exercise involving sprinting, CMJ, change-of-direction tasks, and repeated-sprint ability | 400 mg; capsules; administered 60 min before testing; single dose | DOMS: PRE and 24 h; RPE and HR: POST and 2 min post-exercise | DOMS and RPE [Perceptual]; HR [Physiological]; sport-specific performance [Functional] | CAF had no clear effects on DOMS, RPE, HR, or sport-specific performance. |
| Park et al.  (2008) | Crossover design;  n = 13; both legs/two experimental conditions | University students; 4 men and 9 women; 25.5 ± 3.3 years | Knee-extensor eccentric muscle-damage protocol consisting of 50 eccentric contractions | 6 mg/kg; capsules; administered 30 min before testing; repeated across assessment time points | PRE, 30 min, 1 day, and 2 days after muscle-damaging exercise | MVIC, muscle activation, and electrically evoked force [Functional] | CAF improved strength and muscle activation in the non-damaged muscle but had limited effects on recovery of the damaged muscle. |
| Chen et al.  (2019) | Crossover design;  n = 20;  different treatments at 24 and 48 h | Elite university athletes; 10 men and 10 women; approximately 21 years | Downhill running-induced EIMD performed for 30 min at a −15% gradient and 70% VO₂max | 6 mg/kg; capsules; administered 60 min before testing at 24 and 48 h after EIMD | 24 and 48 h after EIMD; before and after supplementation at each assessment | MVIC, time to exhaustion, and fatigue index [Functional]; DOMS [Perceptual]; CK, blood glucose, and blood lactate [Physiological] | CAF improved MVIC and reduced DOMS, whereas its effects on blood biomarkers varied across outcomes. |
| Maridakis et al.  (2007) | Crossover design; primary analysis n = 9; different treatments at 24 and 48 h | College-aged women;  21.3 ± 1.6 years | Electrically stimulated eccentric quadriceps muscle-damage protocol consisting of 8 × 8 contractions | 5 mg/kg; capsules; administered 60 min before testing at 24 and 48 h after EIMD | 24 and 48 h after EIMD | MVIC [Functional]; pain during eccentric contractions assessed using a VAS [Perceptual] | CAF significantly reduced pain during eccentric contractions but produced only small improvements in MVIC. |
| Ramos-Campo et al. (2019) | Crossover design;  n = 15; CAF/PLA | Trained male athletes;  23.7 ± 8.2 years | Evening 800-m run followed by sleep monitoring and a second 800-m run 24 h later | 6 mg/kg; capsules; administered 60 min before the evening 800-m run; single dose | After the evening 800-m run and during the second 800-m test 24 h later | Objective and subjective sleep outcomes [Sleep]; next-day 800-m and CMJ performance [Functional] | CAF reduced sleep efficiency and increased awakenings, without clearly improving next-day performance. |
| Hurley et al.  (2013) | Crossover design;  n = 9; CAF/PLA | Resistance-trained men;  20 ± 1 years | Biceps resistance exercise performed to failure to induce DOMS | 5 mg/kg; capsules; administered 60 min before the initial exercise bout and repeatedly during recovery | PRE, 24, 48, 72, 96, and 120 h; CK assessed at PRE and 48 h | Soreness and RPE [Perceptual]; range of motion and repetitions completed [Functional]; CK [Physiological] | CAF increased the number of repetitions completed in the final set and reduced soreness during recovery. |
| Miller et al.  (2014) | Crossover design;  n = 6; CAF/PLA | Six male cyclists/triathletes; 27.5 ± 6.9 years | Evening cycling session and time trial followed by PSG-based sleep monitoring | Total dose of 6 mg/kg divided into two doses administered before and during exercise | After evening exercise, during the subsequent sleep period, and at the next-day time trial | Sleep architecture and awakening-related outcomes [Sleep]; time-trial performance [Functional] | CAF improved time-trial performance but may have adversely affected subsequent sleep recovery. |
| Santos-Mariano et al. (2019) | Crossover design;  n = 11; CAF/PLA | Trained male athletes;  18.7 ± 2.7 years | Eccentric-emphasis half-squat exercise consisting of 4 × 12 repetitions at 70% 1RM | 5 mg/kg; capsules; administered before assessments at 24, 48, and 72 h after muscle-damaging exercise | PRE, 24, 48, and 72 h | CK [Physiological]; DOMS and perceived recovery [Perceptual]; MVC, CMJ, and 30-m sprint performance [Functional] | CAF improved CMJ performance at 48–72 h but had limited effects on DOMS, CK, and sprint performance. |
| Filip-Stachnik  et al.  (2022) | Crossover design;  n = 9; CAF/PLA | Trained athletes; 5 men and 4 women; approximately 20–24 years | Evening judo randori training followed by overnight sleep monitoring | 3 mg/kg; capsules; administered 60 min before evening training; single dose | After evening training, during overnight sleep, and on the following morning | Objective sleep outcomes and Karolinska Sleep Diary measures [Sleep]; short recovery–stress state [Perceptual] | CAF did not improve objective sleep or recovery–stress status, and subjective sleep quality was poorer. |
| Ali et al.  (2015) | Crossover design;  n = 10; CAF/PLA | Trained participants;  24 ± 4 years | A 90-min evening intermittent-running protocol simulating soccer activity | 6 mg/kg; capsules; administered 45 min before evening exercise;  single dose | Approximately 15 min post-exercise, the following morning, and additional blood-sampling time points | LSEQ sleep outcomes [Sleep]; next-morning fatigue [Perceptual]; coordination performance [Functional]; blood biomarkers [Physiological] | CAF adversely affected some subjective sleep outcomes but improved certain perceptions related to next-morning alertness. |

*Note: CAF, caffeine; PLA, placebo; PRE, pre-exercise, pre-intervention, or baseline assessment; POST, immediately post-exercise; EIMD, exercise-induced muscle damage; DOMS, delayed-onset muscle soreness; CK, creatine kinase; LDH, lactate dehydrogenase; AST, aspartate aminotransferase; ALT, alanine aminotransferase; CK-MB, creatine kinase myocardial band; MVIC, maximal voluntary isometric contraction; MVC, maximal voluntary contraction; RTD, rate of torque development; CMJ, countermovement jump; RPE, rating of perceived exertion; HR, heart rate; LEF, lower-extremity function; VAS, visual analogue scale; PSG, polysomnography; LSEQ, Leeds Sleep Evaluation Questionnaire; RSA, repeated-sprint ability; 1RM, one-repetition maximum.*

*Outcomes were classified into four domains: Perceptual, including DOMS, soreness, pain, RPE, perceived fatigue, and perceived recovery; Functional, referring to functional performance during recovery and including MVC/MVIC, RTD, CMJ, sprint performance, repetitions completed, time to exhaustion, range of motion, and other performance outcomes; Physiological, including CK, LDH, AST, ALT, CK-MB, myoglobin, blood lactate, blood glucose, oxidative stress markers, and HR; and Sleep, including sleep architecture, sleep efficiency, awakening-related outcomes, subjective sleep outcomes, and sleep questionnaires. For crossover studies, CAF/PLA indicates that the same participants completed both caffeine and placebo conditions; for parallel-group studies, CAF and PLA indicate the numbers of participants assigned to the respective groups. Domain labels describe the recovery outcomes reported in each study; not all listed outcomes contributed to the quantitative synthesis, and only outcomes meeting the quantitative eligibility criteria with sufficient data were included in the meta-analysis.*

Electronic Supplementary Material Appendix S8 ( Risk of Bias in Included Studies )

Table S8A. Risk of bias assessment of the included parallel-group studies

| Study | Recovery domain | D1  (Randomization  process) | D2  （Deviations from intended interventions） | D3  (Missing  outcome data) | D4  （Measurement of the outcome） | D5  （Selection of the reported result） | Overall risk  of bias |
| --- | --- | --- | --- | --- | --- | --- | --- |
| Machado et al. (2009) | Physiological | Some concerns | Low risk | Low risk | Low risk | Some concerns | Some concerns |
| Caldwell et al. (2017) | Functional | Some concerns | High risk | High risk | Some concerns | Some concerns | High risk |
| Bezuglov et al. (2025) | Perceptual | Some concerns | Low risk | Low risk | Some concerns | Some concerns | Some concerns |
| Bezuglov et al. (2025) | Physiological | Some concerns | Low risk | Low risk | Low risk | Some concerns | Some concerns |

Table S8B. Risk of bias assessment of the included crossover studies

| Study | Recovery domain | D1  (Randomization process) | DS  （Period and carryover effects） | D2  （Deviations from intended interventions） | D3  (Missing outcome data) | D4  （Measurement of the outcome） | D5  （Selection of the reported result） | Overall  risk of bias |
| --- | --- | --- | --- | --- | --- | --- | --- | --- |
| AbuMoh’d et al.  （2021） | Physiological | Some concerns | Low risk | Low risk | Low risk | Low risk | Some concerns | Some concerns |
| AbuMoh’d et al.  （2021） | Functional | Some concerns | Low risk | Low risk | Low risk | Low risk | Some concerns | Some concerns |
| Al-Nawaiseh et al.（2022） | Functional | Some concerns | Some concerns | Low risk | Some concerns | Some concerns | Some concerns | Some concerns |
| Al-Nawaiseh et al.（2022） | Perceptual | Some concerns | Some concerns | Low risk | Some concerns | Some concerns | Some concerns | Some concerns |
| Ali et al.  （2015） | Sleep | Some concerns | Low risk | Low risk | Low risk | Some concerns | Some concerns | Some concerns |
| Filip-Stachnik et al.（2023） | Perceptual | Low risk | Some concerns | Low risk | Some concerns | Some concerns | High risk | High risk |
| Filip-Stachnik et al.（2023） | Physiological | Low risk | Some concerns | Low risk | Some concerns | Low risk | High risk | High risk |
| Green et al.  （2018） | Functional | Some concerns | Some concerns | Low risk | Low risk | Some concerns | Some concerns | Some concerns |
| Green et al.  （2018） | Perceptual | Some concerns | Some concerns | Low risk | Low risk | Some concerns | Some concerns | Some concerns |
| Machado et al.  （2008） | Physiological | Some concerns | Some concerns | Low risk | Low risk | Low risk | Some concerns | Some concerns |
| Park et al.  （2008） | Functional | Some concerns | Some concerns | Low risk | Low risk | Low risk | Some concerns | Some concerns |
| Chen et al.  （2019） | Functional | Some concerns | Some concerns | Low risk | Low risk | Some concerns | Some concerns | Some concerns |
| Chen et al.  （2019） | Perceptual | Some concerns | Some concerns | Low risk | Low risk | Some concerns | Some concerns | Some concerns |
| Maridakis et al.  （2007） | Perceptual | Some concerns | Some concerns | Low risk | High risk | Some concerns | High risk | High risk |
| Maridakis et al.  （2007） | Functional | Some concerns | Some concerns | Low risk | High risk | Some concerns | High risk | High risk |
| Ramos-Campo et al.（2019） | Functional | Some concerns | Low risk | Low risk | Low risk | Some concerns | Some concerns | Some concerns |
| Ramos-Campo et al.（2019） | Sleep | Some concerns | Low risk | Low risk | Low risk | Some concerns | Some concerns | Some concerns |
| Hurley et al.  （2013） | Perceptual | Some concerns | High risk | Low risk | Some concerns | Some concerns | High risk | High risk |
| Miller et al.  （2014） | Sleep | Some concerns | Low risk | Low risk | Low risk | Low risk | Some concerns | Some concerns |
| Santos-Mariano et al.（2019） | Physiological | Some concerns | Some concerns | Low risk | Some concerns | Low risk | Some concerns | Some concerns |
| Santos-Mariano et al.（2019） | Perceptual | Some concerns | Some concerns | Low risk | Some concerns | Some concerns | Some concerns | Some concerns |
| Santos-Mariano et al.（2019） | Functional | Some concerns | Some concerns | Low risk | Some concerns | Low risk | Some concerns | Some concerns |
| Filip-Stachnik et al.（2022） | Sleep | Low risk | Low risk | Low risk | Some concerns | Some concerns | Some concerns | Some concerns |

Fig. S2A. Risk-of-bias assessment at the study-by-recovery-domain level for the included parallel-group studies

*Note: The traffic-light plot presents the domain-level and overall risk-of-bias judgments for each study-by-recovery-domain assessment, while the summary bar plot shows the proportions of study-by-recovery-domain assessments rated as low risk, some concerns, or high risk.*

Fig. S2B. Risk-of-bias assessment at the study-by-recovery-domain level for the included crossover studies

***Note:*** *The traffic-light plot presents the domain-level and overall risk-of-bias judgments for each study-by-recovery-domain assessment, while the summary bar plot shows the proportions of study-by-recovery-domain assessments rated as low risk, some concerns, or high risk. For crossover studies, DS denotes bias arising from period and carryover effects.*

Electronic Supplementary Material Appendix S9 ( Heterogeneity estimates )

Supplementary Table S9. Variance components and heterogeneity estimates from the three-level random-effects models

| Outcome domain | No. of effect sizes | No. of studies | τ² Level 3 | τ² Level 2 | *I²* Level 3 (%) | *I²* Level 2 (%) | Total *I²* (%) | 95% prediction interval |
| --- | --- | --- | --- | --- | --- | --- | --- | --- |
| Perceptual recovery | 26 | 7 | 0.0962 | 0.0568 | 37.78 | 22.30 | 60.08 | −0.980 to 0.658 |
| Functional performance during recovery | 30 | 9 | 0.0096 | 0.0000 | 10.28 | 0.00 | 10.28 | −0.444 to 0.028 |
| Physiological recovery | 36 | 5 | 0.0154 | 0.0171 | 8.79 | 9.74 | 18.54 | −0.492 to 0.284 |
| Sleep recovery | 28 | 4 | 0.0716 | 0.0511 | 28.50 | 20.33 | 48.83 | −0.002 to 1.503 |
| Overall recovery, exploratory | 120 | 16 | 0.2055 | 0.0290 | 58.67 | 8.27 | 66.94 | −0.935 to 1.022 |

*Note: Level 3 represents between-study heterogeneity, whereas Level 2 represents heterogeneity among effect sizes within the same study. Variance components were estimated using three-level random-effects models with restricted maximum likelihood estimation. The reported I² values represent the approximate decomposition of total heterogeneity across Levels 2 and 3. The overall recovery model was exploratory. τ² denotes the estimated variance component, and I² represents the proportion of total variability attributable to heterogeneity.*
